# A conserved human CD4^+^ T cell subset recognizing the mycobacterial adjuvant trehalose monomycolate

**DOI:** 10.1172/JCI185443

**Published:** 2024-12-24

**Authors:** Yuki Sakai, Minori Asa, Mika Hirose, Wakana Kusuhara, Nagatoshi Fujiwara, Hiroto Tamashima, Takahiro Ikazaki, Shiori Oka, Kota Kuraba, Kentaro Tanaka, Takashi Yoshiyama, Masamichi Nagae, Yoshihiko Hoshino, Daisuke Motooka, Ildiko Van Rhijn, Xiuyuan Lu, Eri Ishikawa, D. Branch Moody, Takayuki Kato, Shinsuke Inuki, Go Hirai, Sho Yamasaki

**Affiliations:** 1 Department of Molecular Immunology, Research Institute for Microbial Diseases,; 2 Laboratory of Molecular Immunology, Immunology Frontier Research Center (IFReC), and; 3 Laboratory for CryoEM Structural Biology, Institute for Protein Research, Osaka University, Suita, Japan.; 4 Department of Food and Nutrition, Faculty of Contemporary Human Life Science, Tezukayama University, Nara, Japan.; 5 Graduate School of Pharmaceutical Sciences, Kyushu University, Fukuoka, Japan.; 6 Graduate School of Pharmaceutical Sciences, Kyoto University, Kyoto, Japan.; 7 Genome Information Research Center, Research Institute for Microbial Diseases, Osaka University, Suita, Japan.; 8 Respiratory Disease Center, Fukujuji Hospital, Japan Anti-Tuberculosis Association, Tokyo, Japan.; 9 Department of Mycobacteriology, Leprosy Research Center, National Institute of Infectious Diseases, Higashimurayama, Tokyo, Japan.; 10 Division of Rheumatology, Immunity and Inflammation, Brigham and Women’s Hospital, Harvard Medical School, Boston, Massachusetts, USA.; 11 Faculty of Veterinary Medicine, Department of Infectious Diseases and Immunology, University Utrecht, Utrecht, Netherlands.; 12 Department of Medical Biology, Amsterdam University Medical Center, Amsterdam, Netherlands.; 13 Graduate School of Biomedical Sciences, Tokushima University, Tokushima, Japan.; 14 Center for Infectious Disease Education and Research (CiDER), Osaka University, Suita, Japan.; 15 Center for Advanced Modalities and Drug Delivery Systems (CAMaD), Osaka University, Suita, Japan.

**Keywords:** Immunology, Infectious disease, Structural biology, T cell receptor, Tuberculosis

## Abstract

*Mycobacterium tuberculosis* causes human tuberculosis (TB). As mycobacteria are protected by a thick lipid cell wall, humans have developed immune responses against diverse mycobacterial lipids. Most of these immunostimulatory lipids are known as adjuvants acting through innate immune receptors, such as C-type lectin receptors. Although a few mycobacterial lipid antigens activate unconventional T cells, the antigenicity of most adjuvantic lipids is unknown. Here, we identified that trehalose monomycolate (TMM), an abundant mycobacterial adjuvant, activated human T cells bearing a unique αβ T cell receptor (αβTCR). This recognition was restricted by CD1b, a monomorphic antigen-presenting molecule conserved in primates but not mice. Single-cell TCR-RNA-Seq using newly established CD1b-TMM tetramers revealed that TMM-specific T cells were present as CD4^+^ effector memory T cells in the periphery of uninfected donors but expressed IFN-γ, TNF, and anti-mycobacterial effectors upon TMM stimulation. TMM-specific T cells were detected in cord blood and PBMCs of donors without bacillus Calmette-Guérin vaccination but were expanded in patients with active TB. A cryo-electron microscopy study of CD1b-TMM-TCR complexes revealed unique antigen recognition by conserved features of TCRs, positively charged CDR3α, and long CDR3β regions. These results indicate that humans have a commonly shared and preformed CD4^+^ T cell subset recognizing a typical mycobacterial adjuvant as an antigen. Furthermore, the dual role of TMM justifies reconsideration of the mechanism of action of adjuvants.

## Abstract 2

In this paper, we identified a novel effector memoty T cell subset recognizing mycobacterial lipids.

## Graphical abstract

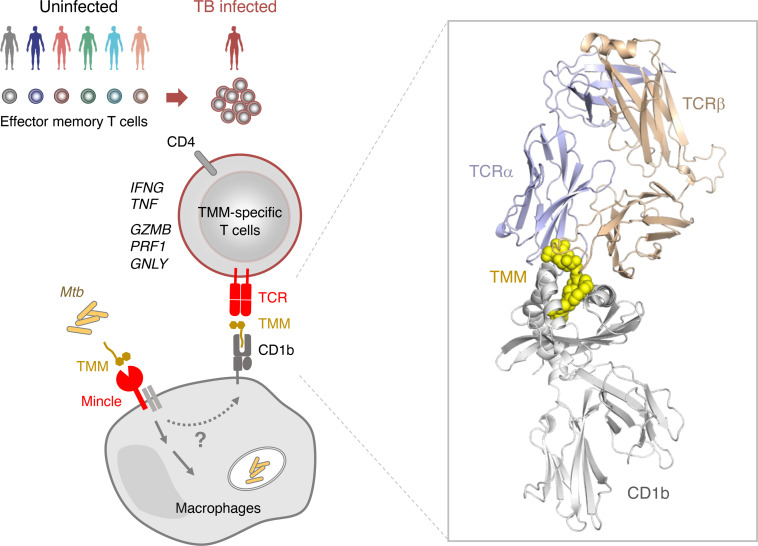

## Introduction

Diseases caused by mycobacteria, including tuberculosis (TB), leprosy, Buruli ulcer, and nontuberculous mycobacterial (NTM) lung disease, rank among the top causes of death and disability worldwide. Mycobacteria are distinguished from other bacteria by a thick cell envelope composed of a unique outer membrane of neutral lipids and glycolipids, which forms the primary barrier against the host and activates host immunity (1). The mycobacterial cell envelope, provided as a mixture of compounds, has been broadly administered in vivo in animals as CFA that can promote a strong vaccine response (2). Yet, a limited understanding of the defined immunogenic components of CFA has to date prevented its therapeutic use as a vaccine adjuvant in humans (3). Recently, studies have identified receptors for some mycobacterial immunogens, which involve *N*-acetyl muramyl dipeptide/nucleotide-binding oligomerization domain-containing protein 2 (MDP/NOD2) and trehalose monomycolate/dimycolate/Mincle (TMM/TDM/Mincle) axes (4–6). However, there remain many mycobacterial lipids for which the receptors and mechanisms of action are not known.

In this study, we sought to identify mycobacterial cell wall components leading to immune activation ex vivo. After demonstrating strong T cell activation, we purified the stimulatory component and established its structure as TMM. T cell activation by TMM was not mediated by Mincle but rather by unique T cell receptor (TCRs) restricted by CD1b. Using TMM-loaded CD1b tetramers and single-cell TCR–RNA-Seq (scTCR-RNA-Seq), we identified a naturally occurring memory T cell population that exists in cord blood and uninfected individuals but is expanded in humans during *Mycobacterium* *tuberculosis* infection. Structural analysis identified unique TCR motifs that are shared across unrelated humans and mediate TMM recognition. Finally, clonotype tracking revealed that TMM-specific T cells produce typical mycobactericidal effectors including granulysin (7), granzyme B (8), IFN-γ (9), and TNF (10, 11) when stimulated with TMM.

## Results

### Identification of mycobacterial lipid–reactive T cells in human PBMCs.

**Figure 1:**
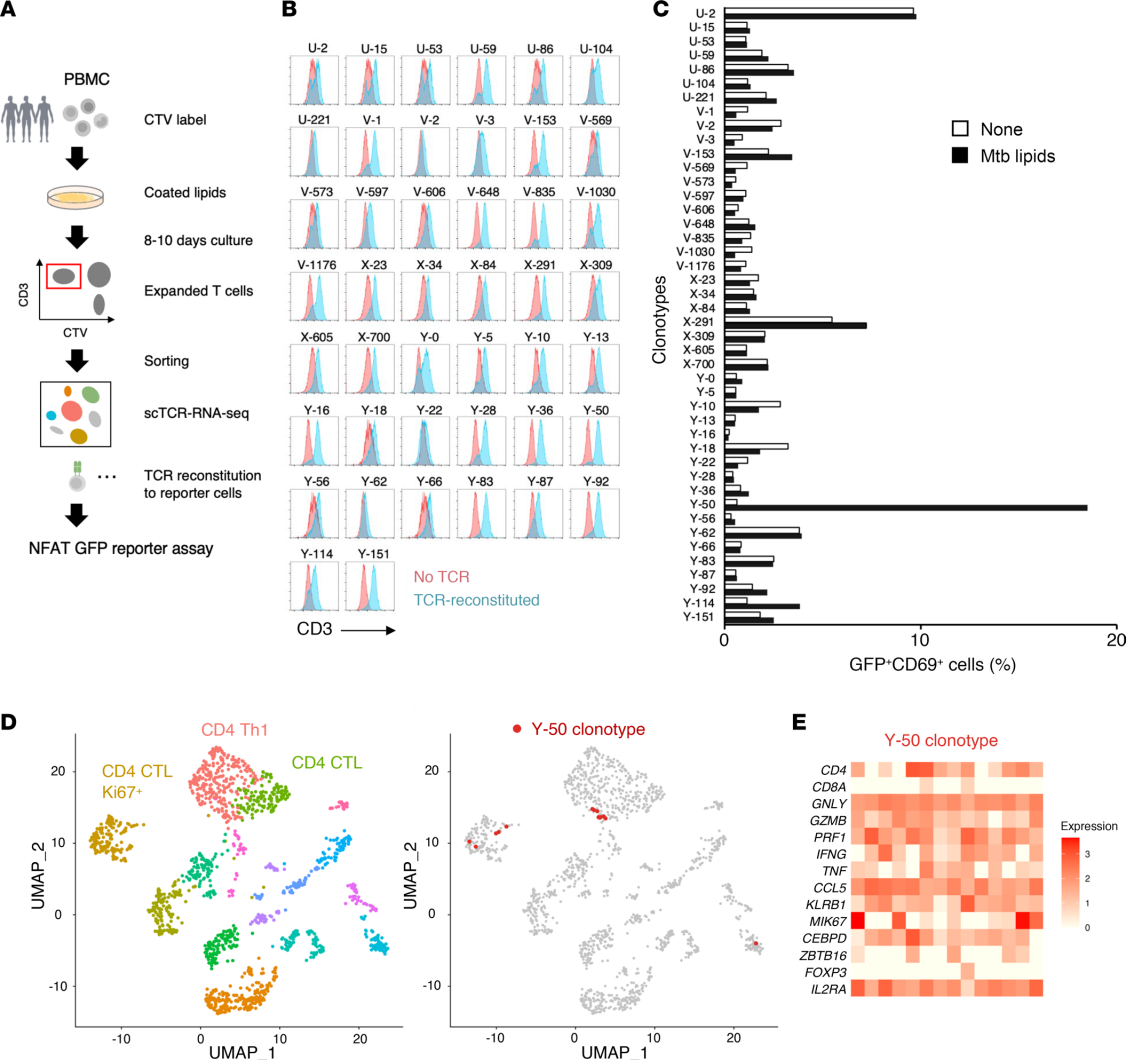
Identification of mycobacterial lipid–reactive T cells. (**A**) Schematic representation of the experimental procedure. Human PBMCs were cultured with plate-coated crude lipids extracted from *M*. *tuberculosis*. The expanded T cells were sorted and analyzed by single-cell TCR-RNA-Seq. Highly expanded CTV^lo^ TCR clonotypes were reconstituted into NFAT-GFP reporter cells to examine the reactivity to *M*. *tuberculosis* lipids. (**B**) Forty-four TCR clonotypes were reconstituted into reporter cells and analyzed for their surface expression using anti-CD3 antibody. (**C**) NFAT-GFP reporter cells (44 clonotypes) expressing each different TCR were stimulated with *M*. *tuberculosis* (Mtb) crude lipids in the presence of PBMCs or cytokine-differentiated monocytes as APCs and, after a 20-hour incubation, analyzed for GFP and CD69 expression. Representative results from 2 independent experiments are shown. (**D**) UMAP plots of T cells expanded in response to *M*. *tuberculosis* lipids (left panel). T cell clones expressing Y-50 clonotype are highlighted by red dots (right panel). CTL, cytotoxic T lymphocytes. (**E**) Heatmap of the gene expression signature of Y-50 cells, with expression of characteristic genes in each cell expressing Y-50 TCR clonotype shown.

As a relatively unbiased and comprehensive way to search for defined immunostimulatory components in the mycobacterial cell envelope, PBMCs from healthy donors were stimulated with total *M*. *tuberculosis* compounds extracted into chloroform/methanol (2:1, vol/vol) and coated onto tissue culture plates (12). On the basis of CellTrace Violet (CTV) dilution, we subjected mycobacterial lipid–responsive T cells to scTCR-RNA-Seq to identify potentially diverse clonotypes along with their effector function and TCR profiles (Figure 1A) (Gene Expression Omnibus [GEO] GSE260931). Among 26,502 detected clonotypes, we selected 52 that are expanded by *M*. *tuberculosis* lipids and reconstituted their TCRαβ pairs in nuclear factor of activated T-cells–GFP (NFAT-GFP) reporter cells (Figure 1A and Supplemental Table 1; supplemental material available online with this article; https://doi.org/10.1172/JCI185443DS1).

After surface expression of the TCR complex was confirmed for 44 clonotypes (Figure 1B), we tested these cells for responses to plate-coated *M*. *tuberculosis* lipids in the presence of cytokine-differentiated human monocytes as antigen-presenting cells (APCs). One clonotype derived from CD4^+^ T cells, Y-50, responded strongly to mycobacterial lipids, based on GFP and CD69 upregulation (Figure 1C). Analysis of scTCR-RNA-Seq data revealed that the Y-50 clonotype was expressed by 14 individual cells within the CD4^+^ T cell clusters (Figure 1D). These cells were characterized by the expression of granzyme B (*GZMB*), perforin-1 (*PRF1*), granulysin (*GNLY*), TNF (*TNF*) and IFN-γ (*IFNG*), regardless of the expression level of *CD4* (Figure 1E and Supplemental Figure 1). Y-50 also expressed innate-like T cell markers, like CD161/killer cell lectin like receptor B1 (*KLRB1*) and CCAAT enhancer binding protein delta (*CEBPD*) (Figure 1E). Some Y-50 cells were also detected in a Ki67^hi^ proliferating CD4^+^ cluster (Figure 1D), in agreement with their CTV^lo^ status used for sorting (Figure 1A). These results suggest that mycobacterial lipid–reactive Y-50–expressing CD4^+^ T cells have an innate and cytolytic signature after ex vivo lipid stimulation.

### TMM activates Y-50 clonotype T cells.

**Figure 2:**
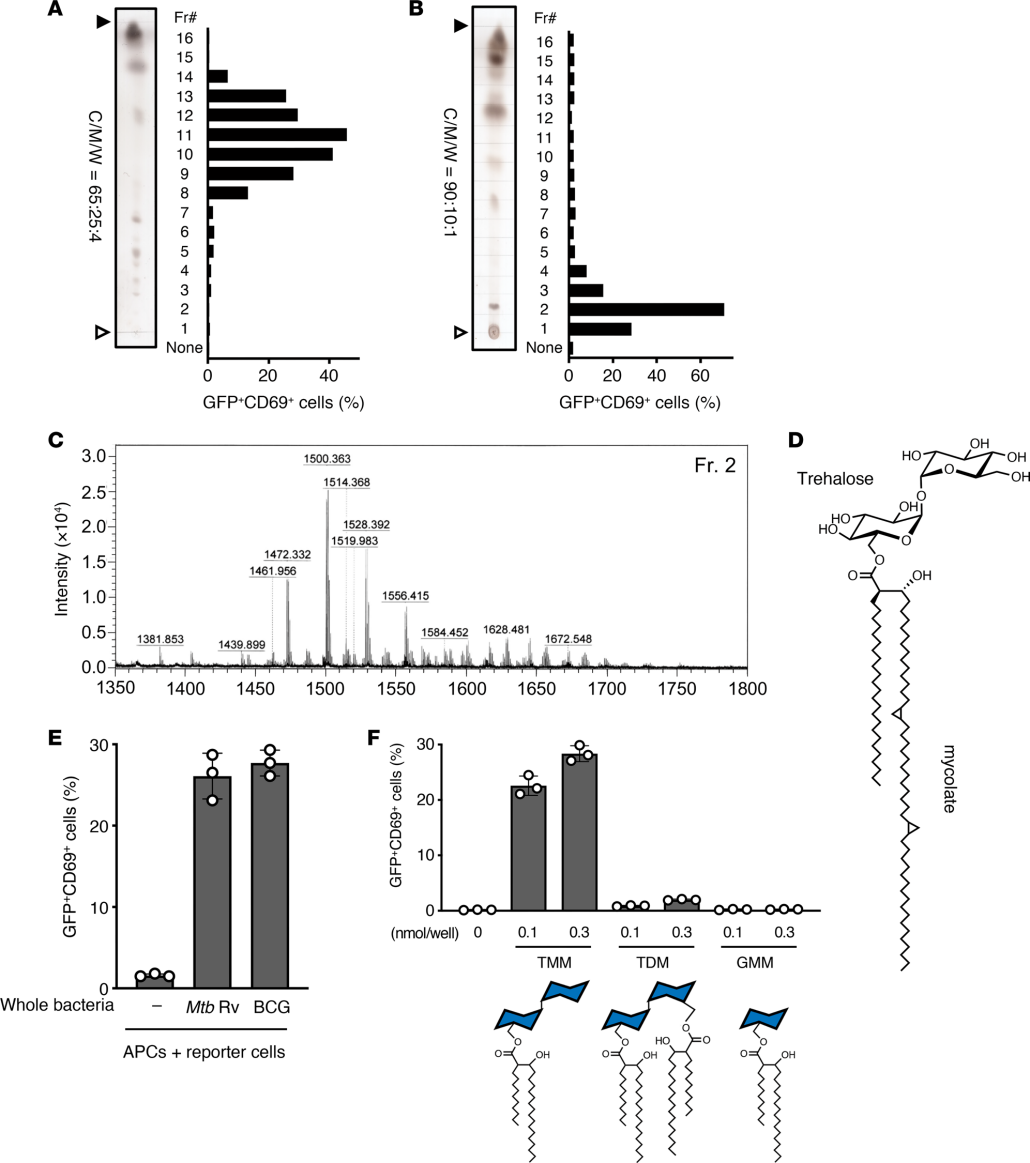
Identification of TMM as a T cell antigen. (**A** and **B**) *M*. *tuberculosis* H37Rv crude lipids were fractionated by HPTLC using chloroform/methanol/water (C/M/W, 65:25:4; vol/vol/vol) (**A**) and 90:10:1; vol/vol/vol (**B**) and stained with copper(II) acetate-phosphoric acid. Y-50 reporter cells were stimulated with each fraction in the presence of APCs and analyzed for GFP and CD69 expression. White and black arrowheads denote the origin and the solvent front, respectively. (**C**) MALDI-TOF MS spectrum of lipid fraction 2 (Fr2). (**D**) The chemical structure of TMM of α-mycolate is shown, and methoxy-mycolate and keto-mycolate are the other major subclasses of mycolate found in *M*. *tuberculosis* TMM. (**E**) Y-50 reporter cells were cocultured with cytokine-differentiated human monocytes preincubated with whole bacteria (heat-killed *M*. *tuberculosis* H37Rv or living BCG) and analyzed for GFP and CD69 expression. (**F**) Y-50 reporter cells were stimulated with the indicated concentration of TMM, TDM, or GMM. Expression of GFP and CD69 is shown in the bar graphs. Schematic ligand structures are shown below. Data are shown as the mean ± SD of triplicate assays (**E** and **F**) and representative results from 2 independent experiments are shown (**A**, **B**, **E**, and **F**).

To identify the lipid stimulus, we separated crude lipids by thin-layer chromatography (TLC) and measured responses to each fraction. We collected 16 fractions and found potent antigen activity (Figure 2A). The active peak shifted to lower retention factor (Rf) values (fraction 2) under more hydrophobic solvent conditions (Figure 2B), suggesting that the antigenic component was probably a moderately polar lipid. We thus analyzed the fraction by matrix-assisted laser desorption/ionization–time-of-flight mass spectrometry (MALDI-TOF MS) (Figure 2C), finding ions that matched and largely overlapped in chain length and saturation patterns with purified TMM from *M*. *tuberculosis* H37 Rv (13) (Figure 2D and Supplemental Figure 2). In addition to purified TMM, Y-50 TCR-expressing reporter cells were also activated by APCs cocultured with *M*. *tuberculosis* H37 Rv and *Mycobacterium* *bovis* bacillus Calmette-Guérin (BCG) (Figure 2E), demonstrating the origin of the stimulus from intact bacteria.

### CD1b restricts TMM recognition by the Y-50 TCR.

TMM is most well known as a major cell wall glycolipid with adjuvanticity that is also a biosynthetic intermediate to TDM, known as cord factor. As TDM and TMM are both known to potently activate myeloid cells through the innate receptor Mincle (12, 14), TMM might activate T cells via Mincle on APCs. However, we found that the Y-50 T cells selectively recognized TMM but not TDM (Figure 2F), suggesting that the response was specific to some aspects of the TMM structure and was not mediated by Mincle.

The major alternative hypothesis was CD1 presentation of TMM to TCRs, as prior studies reported that another mycobacterial glycolipid, glucose monomycolate (GMM), is a CD1-restricted T cell antigen (15). Yet, we observed that GMM was not a Y-50 antigen (Figure 2F), suggesting that TMM might be a new T cell antigen presented by CD1.

**Figure 3:**
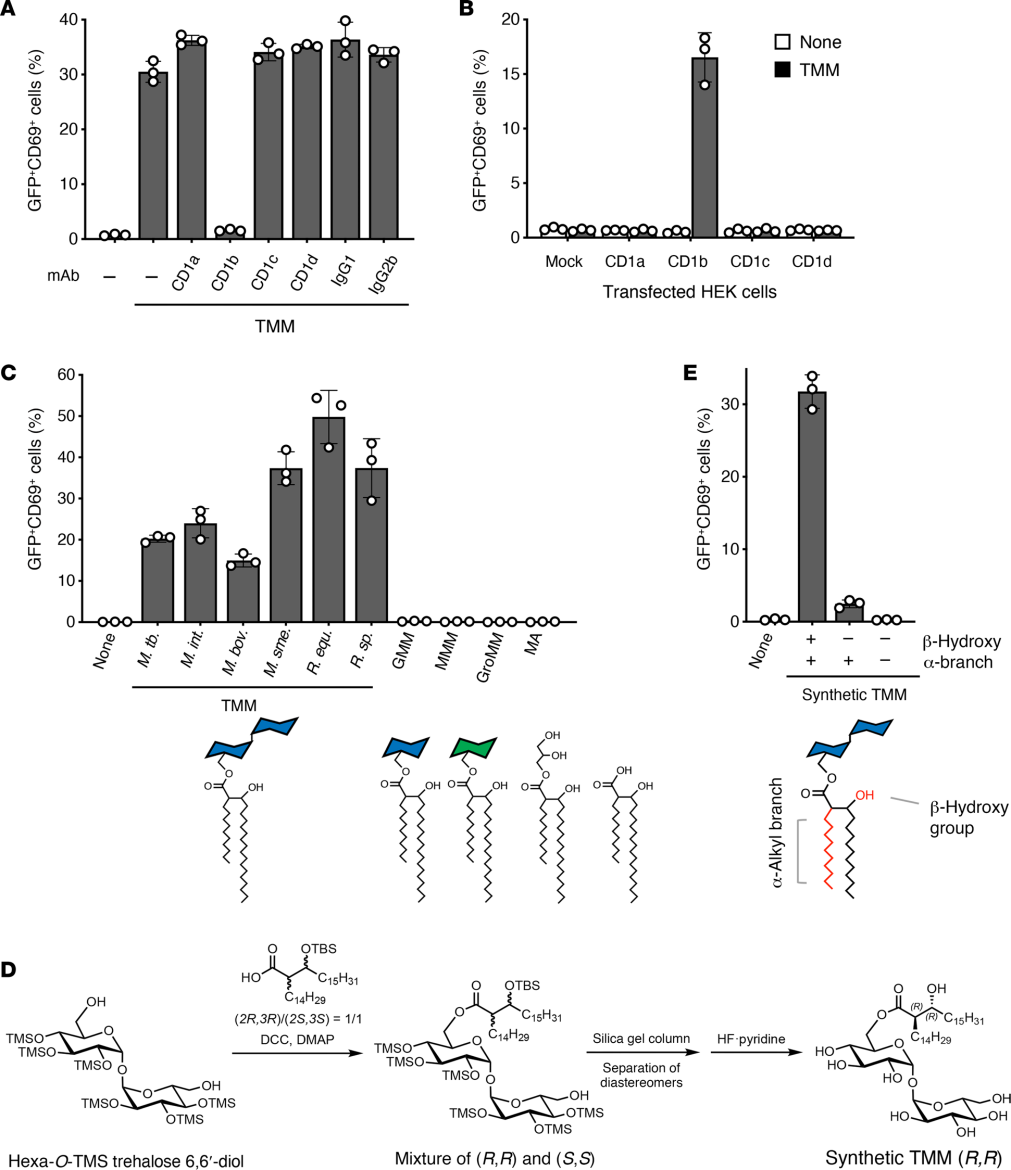
CD1b restricts TMM recognition by Y-50 T cells. (**A**) Y-50 reporter cells were cocultured with cytokine-differentiated human monocytes and TMM (0.3 nmol /well) in the presence of 5 μg/mL anti-CD1a, -CD1b, -CD1c, -CD1d or isotype control antibodies (IgG1 and IgG2b) and analyzed for GFP and CD69 expression. (**B**) The reporter cells expressing Y-50 TCR were stimulated with TMM (1 nmol/well) in the presence of HEK293T cells transfected with human CD1a, CD1b, CD1c, or CD1d. (**C**) Y-50 reporter cells were stimulated with TMM (1 nmol/well) purified from *M*. *tuberculosis* CDC1551, *M*. *bovis* BCG*, M*. *intracellulare, M*. *smegmatis*, *Rhodococcus* *equi*, and *R*. *sp* 4306. Also, GMM, MMM, GroMM, and MA were tested in the presence of human CD1b-expressing DC2.4 cells (CD1b-DC2.4). (**D**) Scheme for synthetic TMM. (**E**) Y-50 reporter cells were stimulated with synthetic TMM harboring a β-hydroxy group and α-branched alkyl chains or synthetic analogs lacking hydroxy (–, +) or both moieties (–, –) in the presence of CD1b-DC2.4 cells as APCs. Data are shown as the mean ± SD of triplicate assays, and representative results from 2 independent experiments are shown (**A**–**C** and **E**).

Given that all 4 types of human CD1 antigen–presenting molecules can present lipids (16), we examined the effect of blocking antibodies against human CD1a, CD1b, CD1c, and CD1d and found that only anti-CD1b selectively suppressed TMM-induced activation of Y-50 reporter cells in the presence of APCs (Figure 3A). Conversely, ectopic expression of CD1b on HEK293T cells conferred Y-50 TCR reactivity to TMM (Figure 3B). Thus, only CD1b is necessary and sufficient for the presentation of TMM to the Y-50 TCR.

### Clone Y-50 broadly recognizes TMM from various mycobacterial species.

TMM is produced broadly among mycobacterial species (17). To further characterize the selectivity of antigenic lipids recognized by the Y-50 TCR, we purified TMM possessing different lipid lengths from *Mycobacterium* *intracellulare* and *Mycobacterium* *smegmatis* (C60-C88) as well as *Rhodococcus* species with shorter mycolate moieties (C28-C36), and found that all showed antigenic activity (Figure 3C). However, other related mycolyl lipids that varied in the head group moiety — GMM, mannose monomycolate (MMM), glycerol monomycolate (GroMM), and free mycolic acid (MA) (18, 19) — lacked antigenicity (Figure 3C). Thus, the T cell reactivity identified here was new, and the head group composed of the trehalose disaccharide was required for Y-50 TCR recognition.

To exclude the possibility of contaminants or mitogens in natural TMM preparations, we carried out complete synthesis of TMM, starting with hexa-*O*-TMS trehalose 6,6′-diol (Figure 3D). Synthetic TMM also induced T cell activation, formally ruling in this structure as an antigen (Figure 3E). However, other synthetic TMM analogs lacking an α-branched alkyl chain or a β-hydroxy group did not activate (Figure 3E). Thus, while Y-50 broadly recognizes TMM mycolate chains of varying length present across mycobacterial species, it discriminates the chemical features that define mycobacterial TMM.

### Cationic residues in Y-50 TCR are critical for TMM recognition.

**Figure 4:**
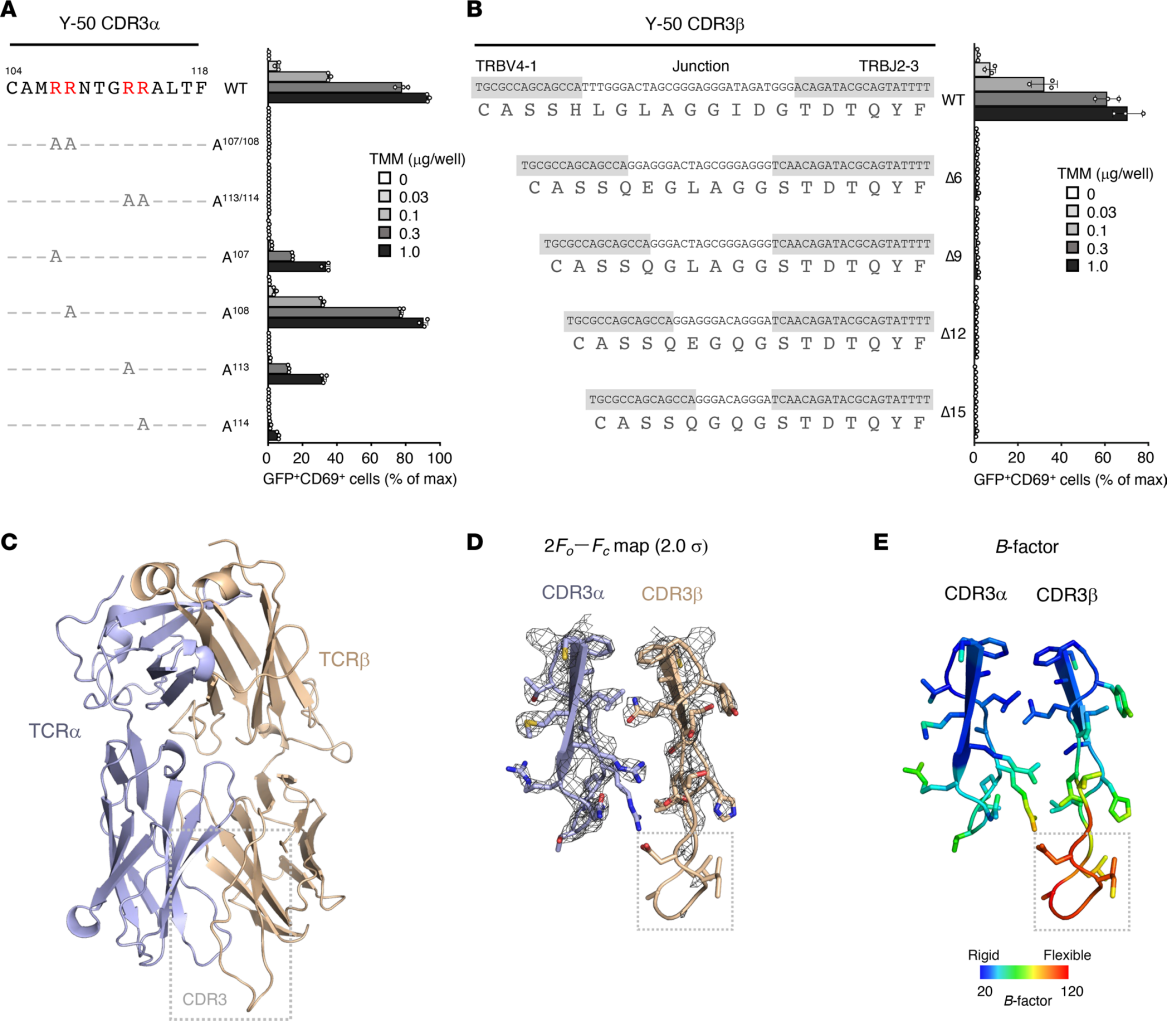
Mutagenesis and structural analysis of TMM-reactive TCR. (**A**) The amino acid sequences of Y-50 CDR3α. Arginine residues which were mutated to alanine are shown in red. TMM reactivities of each mutant are shown as a percentage of the maximum (max) response induced by plate-coated anti-CD3 Ab. The number of amino acids is shown in accordance with the ImMunoGeneTics (IMGT) definition (https://imgt.org/IMGTScientificChart/). (**B**) Nucleotide and amino acid sequences of the Y-50 TCR CDR3β region and its junction deletion mutants (Δ). D region and N or P nucleotide sequences that constitute junctional sequences are unshaded. Cells were stimulated as indicated in **A**. (**C**) Crystal structure of the Y-50 TCRαβ heterodimer (PDB: 8XUB). The main chains of TCRα and β are shown in violet and brown, respectively. CDR3αβ regions are boxed. (**D** and **E**) A 2*Fo*-*Fc* map contoured at 2.0 σ (**D**) and *B*-factor diagram (**E**) of CDR3αβ are shown as gray mesh and color gradient, respectively. Junction regions of CDR3β are boxed. Data are shown as the mean ± SD of triplicate assays (**A** and **B**), and representative results from 2 independent experiments are shown.

A striking characteristic of Y-50 TCRα is the presence of 4 positively charged arginine residues (R107, R108, R113, and R114) within the CDR3α region (Figure 4A). To investigate the contribution of these residues to TMM recognition, we introduced alanine mutations and evaluated their effects using TCR-reconstituted reporter cells. TCRα containing alanine substitutions at R107, R113, showed impaired reporter activity, whereas R108A had no effect (Figure 4A).

Compared with the average CDR3β (14.4 residues) (20), the Y-50 CDR3β was much longer and encoded by 20 residues (Figure 4B). To assess the contribution of amino acid insertion during VDJ recombination to antigen recognition, we engineered 4 shorter Y-50 TCRβs lacking residues within the junctional region. None of these TCRβ mutants recognized TMM (Figure 4B), suggesting that certain aspects of this long CDR3β are required for the recognition of TMM by the Y-50 TCR.

### Structural characterization of the Y-50 TCR.

The cationic and long loop motifs suggested a TCR binding mechanism controlled by electrostatic interactions and a flexible TCR surface. To gain structural insight into the Y-50 TCR, a soluble TCRαβ was constructed for crystallization. We obtained a crystal structure of the TCRαβ complex (Protein Data Bank [PDB]: 8XUB) that diffracted to a resolution of 2.5 Å (Figure 4C and Supplemental Table 2). Three CDR3α arginine residues that were found to be critical for TMM recognition were facing toward the TCRβ, whereas R108, which was dispensable, was oriented away from the TCRα-β interface (Supplemental Figure 3A). The electron density of the TCRαβ was clear except for the CDR3β loop region, implying that the extra-long CDR3β loop may be highly flexible, as hypothesized (Figure 4, D and E).

### Determination of the ternary complex structure of Y-50 TCR-TMM-CD1b.

**Figure 5:**
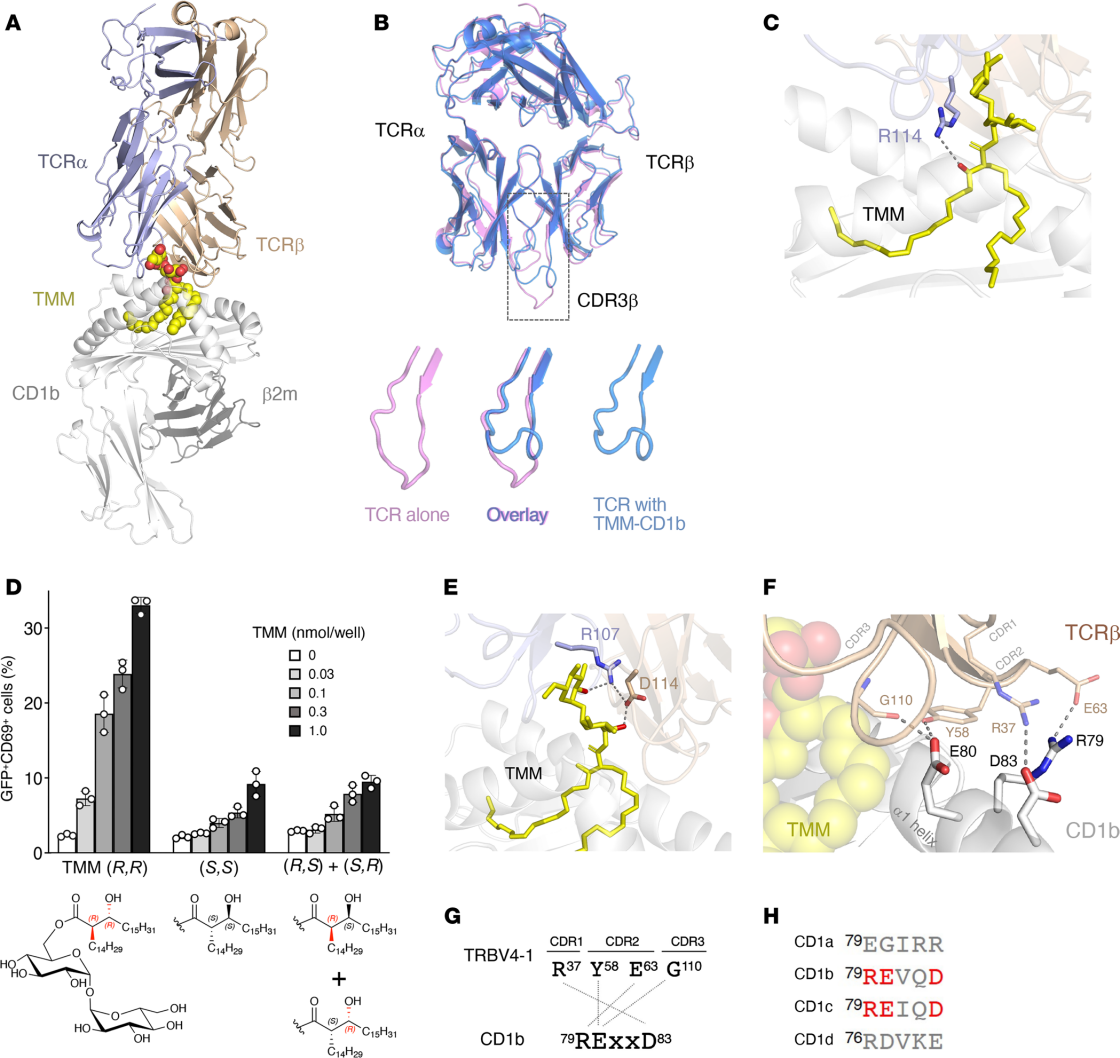
Ternary complex structure of Y-50 TCR-TMM-CD1b. (**A**) Overall structure of the Y-50 TCR-TMM-CD1b complex. The main chains of TCRα, TCRβ, and CD1b are shown. TMM is presented as yellow spheres. (**B**) Upper panel: Superimposition of the structure of Y-50 TCR alone (PDB: 8XUB) (pink) and Y-50 TCR bound to TMM-CD1b (PDB: 8ZOX) (blue) . Lower panels: CDR3β regions (boxed area in the upper panel) magnified. (**C**) Close-up view of TMM (*R,R*) and the side chain of R114 within CDR3α. The β-hydroxy group of TMM is shown in red. (**D**) Y-50 reporter cells were stimulated with the natural configuration of synthetic TMM (*R,R*) or non-natural stereoisomers (*S,S*) or (*S,R+R,S*) in the presence of CD1b-DC2.4 and analyzed for GFP and CD69 expression. The stereoisomer structures are shown below (*R*, red; *S*, black). Data are shown as the mean ± SD of triplicate experiments, and a representative result from 2 independent experiments is shown. (**E**) Close-up view of TMM (*R,R*) and the side chain of R107 (CDR3α) and D114 (CDR3β). Hydroxy groups of TMM that formed hydrogen bonds to the TCR side chains are shown in red. (**F**) Close-up view of the side chains of R79, E80, and D83 in CD1b that interact with the side chains of R37 (CDR1β), Y58 and E63 (CDR2β), and G110 (CDR3β). (**G**) Multi-bonded interaction of the CD1b RExxD motif and TRBV4-1 residues. Individual interaction is shown by dotted lines. (**H**) Conservation of the RExxD motif in human CD1b and CD1c. The amino acid sequences of CD1a (NP_001307581), CD1b (NP_001755.1), CD1c (NP_001756.2), and CD1d (NP_001306074) are aligned. Numbers indicate the amino acid position of the mature peptide (excluding signal peptide).

However, solving the definitive recognition mechanism required a ternary structure, so we conducted cryo–electron microscopy (cryo-EM) analysis. Recombinant CD1b was refolded with synthetic TMM (Supplemental Figure 3B) and incubated with soluble Y-50 TCRαβ. The cryo-EM map of the ternary complex was successfully reconstructed to a resolution of 3.18 Å (PDB: 8ZOX) (Figure 5A, Supplemental Figure 3, C–E, Supplemental Table 3, and Supplemental Video 1). In the area between TCRαβ and CD1b, we observed a clear density map that exactly overlapped with the chemical structure of TMM (Supplemental Figure 3F). TMM lipid chains were buried inside CD1b pockets, like GMM (21), with its sugar head group exposed toward the TCR (Supplemental Figure 3G). TCRαβ tightly contacted with its protruding bulky trehalose moiety through CDR3 regions (Supplemental Figure 3G).

This observation was supported in detail by the superimposition of the structure of the Y-50 TCR alone (Figure 4C, PDB: 8XUB) with its structure within the ternary complex (Figure 5A, PDB: 8ZOX). The positions of backbone Cα atoms in both structures largely overlapped; however, the location of the long CDR3β region was noticeably shifted upon TMM-CD1b contact (Figure 5B). Whereas the CDR3β loop hung “downward” in the structure without antigen, it was “drawn up” like a curtain to allow the recognition of TMM presented by CD1b (Figure 5B), providing detailed insight into how the long CDR3β creates the flexible TCR interface with the CD1b-TMM complex by avoiding steric hindrance.

We next determined the mode of specific TMM recognition by Y-50 TCR. The experimentally observed functional importance of cationic CDR3α residues (Figure 4A) was explained by the ternary structure. R114 of the TCRα chain formed a hydrogen bond with the β-hydroxy group of TMM (Figure 5C), likely explaining both the strong effects of alanine mutation (Figure 4A) and the altered recognition of TMM lacking the β-hydroxy group (Figure 3E). To further test the significance of this interaction, we synthesized TMM stereoisomers that differed in the stereochemistry of the acyl group (Supplemental Figure 4). Y-50 TCR recognized natural TMM (*R,R*) but not non-natural isomers (Figure 5D). Overall, the Y-50 TCR is highly specific for the natural stereoconfiguration of the TMM lipid moiety through interaction with the cationic residue R114.

Trehalose is a diglucose. Another critical cationic residue, TCRα R107, formed a hydrogen bond with the hydroxy group at the C-2′ atom of the distal glucose in trehalose, whereas the proximal glucose interacted with TCRβ D114 through the hydroxy group at the C-4 atom (Figure 5E). Furthermore, α^R107^ or β^D114^ formed a salt bridge (Figure 5E). Thus, Y-50 TCRα and TCRβ may cooperate for TMM recognition by interacting with distinct epitopes. In line with this interpretation, Y-50 TCR recognition of TMM was impaired in α^R107A^ or β^D114A^ single mutation and more severely in double mutation (α^R107A^-β^D114A^) (Supplemental Figure 5A).

These structural analyses further revealed the molecular basis by which the characteristic features of Y-50 TCR recognize the TMM-CD1b complex. Alanine scanning functionally confirmed this mode of antigen recognition and found additional critical residues that are involved in the interaction with CD1b (Supplemental Figure5, B–D). For example, TCRβ G110 was also important (Supplemental Figure 5D) due to an interaction with E80 of CD1b (Figure 5F). In addition to CDR3β, other regions derived from the TRBV4-1-encoded TCR Vβ chain also bound the surface of CD1b itself. A positively charged R37 in CDR1β formed a salt bridge with a negatively charged D83 of CD1b. CDR2β also interacted with the CD1b α1 helix through a salt bridge (E63-R79) and a hydrogen bond (Y58-E80) (Figure 5, F and G, and Supplemental Video 2). Thus, the TRBV4-1-encoded TCR Vβ loops interact with a triply charged patch on CD1b defined as a ^79^RExxD^83^ sequence in an antigen-independent manner (Figure 5G). This charged patch can explain the preferential usage of TRBV4-1 in CD1b- and also CD1c-restricted TCRs (22–24), as an identical ^79^RExxD^83^ motif is also found in CD1c, but not other human CD1 isoforms (Figure 5H).

### Characterization of TMM-specific T cells in the periphery using tetramers.

**Figure 6:**
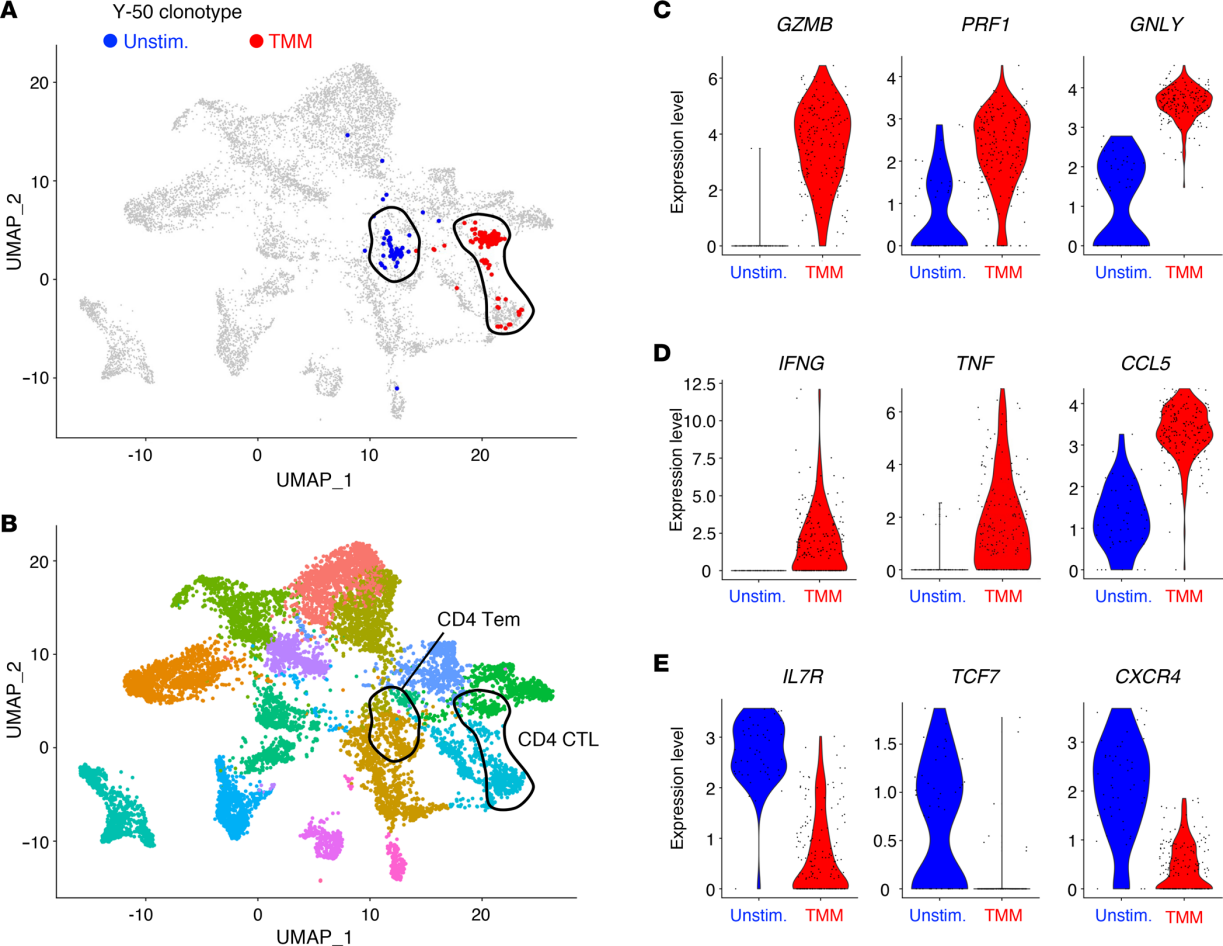
Functional maturation of TMM-reactive T cells upon TMM stimulation. (**A** and **B**) Cluster shift of the Y-50 clonotype before and after TMM stimulation. T cells expressing the Y-50 clonotype defined by scTCR-RNA-Seq are overlaid (**A**) on a UMAP plot of PBMCs from donors including the donor sample used in Figure 1 (**B**), as described in Methods. Tem, effector memory T cells; CTL, cytotoxic T lymphocytes. (**C**–**E**) Differentially expressed genes in Y-50 T cells upon TMM stimulation. Violin plots show the expression of representative genes encoding cytotoxic effector molecules (**C**), proinflammatory cytokines and chemokines (**D**), and stemness-related molecules (**E**). Unstim., unstimulated.

Key questions relating to whether preprimed memory cells recognizing CD1b exist in humans, as well as their potential effector function in the periphery without in vitro expansion, were largely unknown. We therefore sought to generate TMM-loaded CD1b tetramers and combine them with single-cell analysis. These TMM-CD1b and unloaded control tetramers were validated by binding to cell lines expressing TCRs and TMM-stimulated human PBMCs (25) (Supplemental Figure 6). First, we investigated gene expression profiles of freshly isolated and TMM-stimulated T cells bearing Y-50 TCR using scTCR-RNA-Seq. Y-50 T cells were separated into different clusters before and after stimulation in a uniform manifold approximation and projection (UMAP) plot (Figure 6A), implying that gene expression signatures were altered by antigen stimulation. Before ex vivo stimulation, Y-50 T cells expressed typical effector memory markers, such as CD44 (*CD44*), IL-7 receptor α chain (*IL7R*), and integrin β_1_ (*ITGB1*) (Figure 6B and Supplemental Figure 7, A and B), suggesting that resting TMM-specific T cells exist as naturally occurring memory T cells, which is the characteristic feature of NKT and other innate T cells (26). Upon TMM stimulation, Y-50 T cells moved to the cluster characterized by the expression of cytotoxic and bactericidal effector genes (*GZMB*, *PRF1*, *GNLY*) (7, 8) and anti-mycobacterial protective cytokines and chemokines (*IFNG*, *TNF*, *CCL5*) (27, 28), whereas the expression levels of stemness-related molecules, such as *IL7R*, *TCF7*, and *CXCR4*, were downregulated (Figure 6, C–E). These results suggest that Y-50 T cells are innate-like T cells exhibiting anti-mycobacterial potential in the periphery, whose effector signature is markedly enhanced upon antigen stimulation.

### TMM-specific T cells with similar TCR motifs are shared across humans.

**Figure 7:**
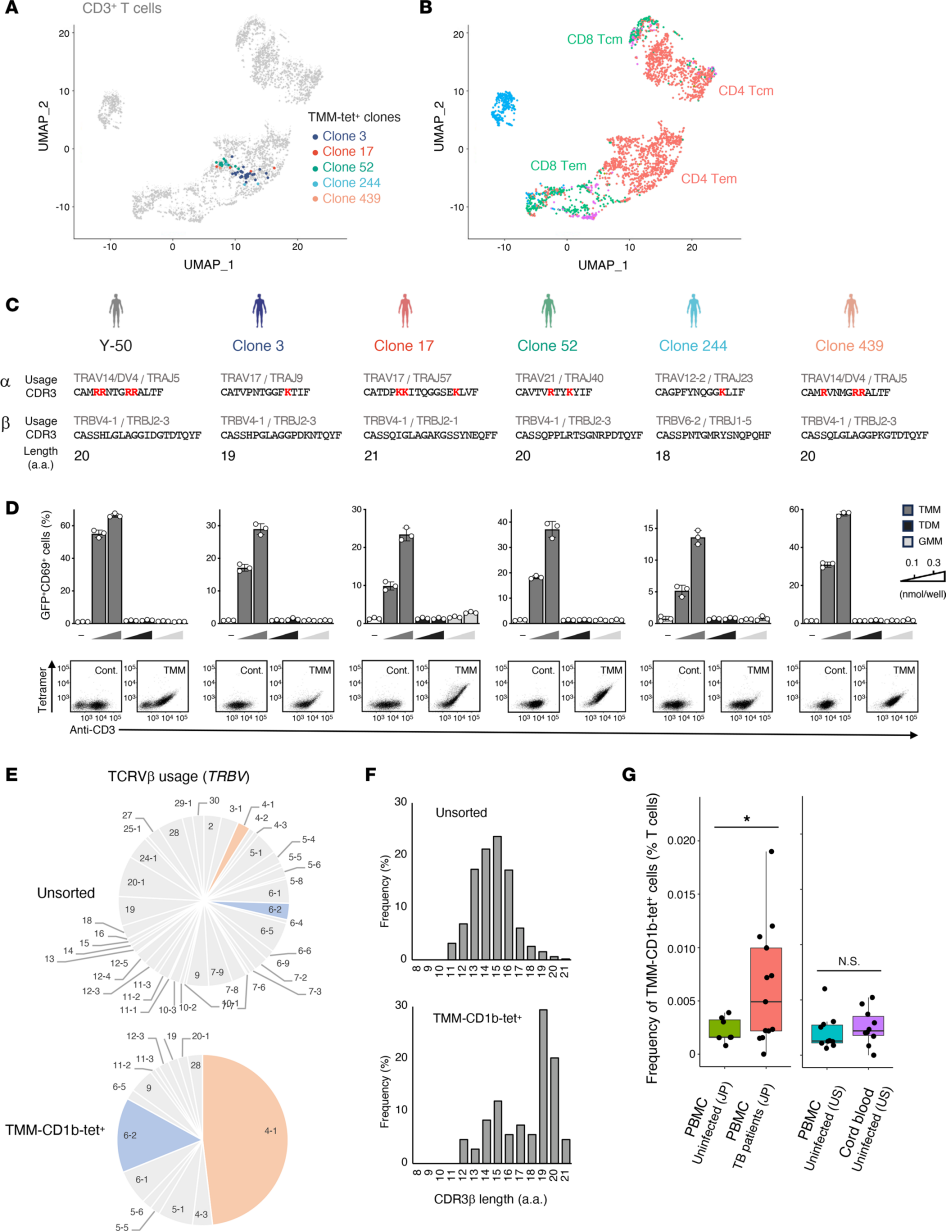
TMM-specific T cells with similar characteristics are shared among individuals. (**A** and **B**) Frequent TMM-specific clonotypes identified by TMM-CD1b-tetramer sorting and scTCR-RNA-Seq are overlaid (**A**) on a UMAP plot of all TMM-tetramer–sorted T cells and unsorted CD3^+^ T cells from 13 healthy donors (**B**). Three clones were detected from different individual donors; 2 clones (clones 17 and 439) were from another donor. CD4^+^ Tem, CD4^+^ effector memory T cells; CD4 Tcm, CD4^+^ central memory T cells. Naive T cells were rare within TMM-tetramer^+^ cells and were not clustered on the UMAP. (**C**) TCR usages, CDR3 sequences, and length of the CDR3β region of the clonotypes detected in **A**. (**D**) Each clonotype was reconstituted into reporter cells and analyzed for TMM, TDM, and GMM reactivity using CD1b-DC2.4 as APCs. Data are shown as the mean ± SD of triplicate assays, and representative results from 2 independent experiments are shown. Reporter cells were stained with PE-conjugated endo-CD1b (Cont.) or TMM-CD1b (TMM) tetramers and anti-CD3 antibodies. Y-50 TCR is shown as a control. (**E** and **F**) Frequency of TCRVβ usage (**D**) and length of the CDR3β region (**E**) of unsorted or the top 27 TMM-CD1b tetramer^+^ T cell clonotypes. (**G**) PBMCs from Japanese donors (uninfected donors, *n* = 7; patients with TB, *n* = 13) or PBMCs (*n* = 10) and cord blood cells (*n* = 10) from uninfected US donors were stained with PE-conjugated TMM-loaded CD1b tetramer, APC-conjugated CD1b-endo tetramer and anti-CD3 antibody. The percentages of TMM-CD1b tetramer^+^ and endo-CD1b tetramer^–^ populations in CD3^+^ T cells are shown (TMM-CD1b-tet^+^). Medians are indicated with horizontal bars. *P < 0.05, by unpaired, 2-tailed Welch’s *t* test.

To examine whether TMM-specific T cells are shared across genetically unrelated individuals, we sorted TMM-CD1b-tet^+^ cells from fresh PBMCs obtained from additional donors and examined their characteristics by scTCR-RNA-Seq. We found that TMM-CD1b-tet^+^ clonotypes from unrelated donors were mainly localized within the CD4^+^ effector memory clusters (Figure 7, A and B), which expressed CD44, the IL-7 receptor, or integrin β_1_ but not the homing receptor CCR7 (Supplemental Figure 7C), similar to unstimulated Y-50 T cells (Supplemental Figure 7B). TCRs expressed by these clonotypes were reconstituted in reporter cells, and their specific reactivities to TMM was confirmed (Figure 7, C and D). Importantly, the sequences of these TCRs revealed them not be identical to Y-50, but they possessed similar characteristics, including positively charged CDR3α sequences, biased TCR Vβ that is encoded by TRBV4-1, and long CDR3β sequences (Figure 7C). Furthermore, these trends could be seen in a comprehensive TCR analysis of TMM-CD1b tetramer–sorted T cells (Figure 7, E and F). Thus, TMM and CD1b-reactive T cells showed clear evidence for conserved features across numerous clonotypes from different donors, constituting a new public TCR-antigen linkage that establishes a donor-unrestricted T cell subset in humans (29).

### Quantification of TMM-specific T cells during M. tuberculosis infection.

Finally, we used tetramers to examine the frequency of TMM-specific T cells in PBMCs from uninfected and donors with active TB, who were recruited consecutively on the basis of smear-positive and *M*. *tuberculosis* culture–positive sputum samples (Supplemental Table 4). We detected TMM-CD1b-tet^+^ T cells in most uninfected donors, consistent with prior experiments of single-cell or tetramer-based outcomes (Figures 6 and 7), further suggesting that these cells are preformed, innate-type T cells, as these frequencies are similar to those of other unconventional T cells (30). The frequency was significantly increased in patients with active TB (Figure 7G), suggesting that these T cells may react to TMM during mycobacterial infection in the host. Since these samples were from healthy Japanese donors who had received BCG vaccination, we also examined PBMCs and cord blood cells from healthy donors from North America, where the BCG vaccine is no longer widely given. The frequency of tetramer^+^ T cells was comparable among all 3 uninfected groups (Figure 7G), indicating that TMM-specific T cells were present in naive donors and developed without exposure to BCG or other environmental antigens.

## Discussion

A basic paradigm for adaptive MHC-restricted T cells is that naive cells are primed by antigen to differentiate into memory T cells that persist in elevated numbers with memory markers in the periphery. In contrast, CD1d-restricted invariant NKT cells express memory markers in the absence of defined antigenic stimulation and functionally circulate in larger numbers and respond rapidly as a cohort to antigen challenge (26, 31). The extent to which human group 1 CD1-restricted (CD1a-, CD1b-, and CD1c-restricted) T cells behave as naive or memory T cells in the periphery, remains poorly understood, owing mainly to technical challenges in directly addressing these questions in humans and limited tractable infection models that express CD1a, CD1b, and CD1c (which are lacking in mice) (32). For example, prior studies have emphasized long-term in vitro–cultured T cell clones (7, 15, 18, 33) or indirect detection of T cells by activation assays (34, 35) rather than tetramer-based scTCR-RNA-Seq analysis. Through the discovery of TMM as a T cell antigen, the generation of CD1b-TMM tetramers applied across unrelated donors, the identification of new binding motifs, and the structural dissection of lipid and nonlipid interaction by a cryo-EM ternary complex, this study advanced our understanding of human pathogen–specific T cell responses in the CD1b system.

Our key findings were the identification of mycobacteria-specific TCRs in peripheral T cells without infection and the rapid induction of protective effectors by stimulation ex vivo. IFN-**γ** and TNF are canonical antimycobacterial cytokines produced from CD4^+^ Th1 cells (9–11). Recently, granulysin, granzyme B, and perforin secreted from CD8^+^ T cells were also recognized to be important for protection against mycobacterial infection (27, 36). TMM-specific T cells are a unique cell subset that rapidly upregulated all of these effectors simultaneously upon antigen stimulations. While host protection was difficult to demonstrate directly in human experimental systems, this evidence supports the notion that TMM-reactive T cells express a host protective effector function.

Although TMM-reactive T cells expressed CD4, this coreceptor seems dispensable for the recognition of the CD1b-TMM complex, as the reporter cells used in our assay did not express human CD4. However, we cannot fully exclude the possibility that, like conventional T cells, CD4 is required for those T cells to be selected by MHC class II. Clinically, the well-known susceptibility of HIV-infected patients to TB resulting from the reduction of CD4^+^ T cells (37) might also be partly due to the loss of CD4^+^ TMM-specific T cells.

Known patterns of TMM biosynthesis and expression support plausible scenarios for TMM antigen function during infection. TMM is expressed by most mycobacterial species and is used for further biosynthesis of other cell wall components, including arabinogalactan and TDM (38). Unlike TDM, which is downregulated in mycobacteria upon infection of the host, the level of TMM is relatively constant (39). Compared with GMM, TMM may be resistant to stresses such as oxidation because the reducing ends of both glucoses are occupied. Thus, given the importance of TMM for multiple stages of the mycobacterial life cycle, the presence of T cells that recognize TMM with various lipid chain lengths plausibly could allow effective induction of responses to a broad spectrum of mycobacteria.

Taking advantage of the direct detection by tetramers and single-cell analysis, we provide several lines of evidence that TMM-specific T cells exist before the host is exposed to mycobacteria, as they were detected in random blood donors, as well as non-TB or non-BCG vaccinated donors and even in cord blood cells. However, as contrasted with NKT and MAIT cells, *PLZF* was not highly expressed in TMM-specific T cells, so it is unclear whether they were selected by DP thymocytes like other innate-like T cells (40, 41). Even assuming the involvement of CD1b for selection, the selecting ligand(s) is unclear. It is also possible that the intrinsic affinity of TRBV4-1 to CD1b patch might allow less ligand-dependent positive selection.

Determination of the ternary structure of TMM-specific TCR provides similarities and differences in the mode of glycolipid recognition with previously reported glycolipid-specific T cells (21). CDR3α loop regions of both TCRs interact with β-hydroxy group of GMM and TMM, which is a defining chemical feature of foreign mycolic acids as contrasted with self fatty acids, which allows T cells to discriminate the natural configuration of mycolyl lipids. Compared with the monosaccharide in GMM, 2 sugar moieties of TMM interacted more extensively with TCR residues, likely determining T cell antigen specificity (Supplemental Video 3). Furthermore, long CDR3β region uniquely found in TMM-specific TCRβ and its demonstrated compression in the ternary structure show how the TCRβ chain moves upward to accommodate the bulky TMM head group presented by CD1b.

Biased usage of TRBV4-1 has been reported for CD1b-restricted T cells from blood (22, 23) and TB pleural effusions (42), suggesting that such TCRs are clinically relevant to host response in TB disease and could be biomarkers of *M*. *tuberculosis* infection. However, a detailed mechanism underlying this preference was unclear, as the ternary complex structure of TRBV4-1^+^ TCR with CD1-bound antigens had never been solved. Our cryo-EM structure provides direct evidence for the presence of an antigen-independent “patch” by which TRBV4-1-encoded residues interact with CD1b. Conservation of this motif among CD1b and even CD1c, but not in other human CD1 molecules, may support the high frequency of TRBV4-1 in CD1b- and CD1c-restricted T cells (24). The first identification of CD1b/c “patch” implies the presence of any other motifs for known biased TCR Vβ genes, such as TRBV7-9^+^ T cells restricted by human CD1c (43) or CD1d, which are associated with Crohn’s disease (44–46).

TMM is recognized by the pattern recognition receptor Mincle (12). Thus, this mycobacterial lipid represents a “dual ligand” that can activate both pattern recognition receptors (PRRs) and TCRs, so our data suggest that TMM can act simultaneously as a pathogen-associated molecular pattern (PAMP) and a T cell antigen, respectively. Freund’s adjuvant cannot be used as a human therapeutic due to its bacterial origin and undefined mechanism. However, chemically defined, dual-acting molecules like the synthetic TMM studied here could be promising therapeutic options, as both adjuvant and antigen, to prevent various diseases caused by mycobacterial species. In addition to TB, NTM lung disease is one of the most urgent targets, as cases are dramatically increasing and current drug treatments are ineffective (47). TMM from NTM species are also – and more strongly – recognized by TMM-specific T cells. Furthermore, the potent activity and higher hydrophilicity of short-chain TMMs could be advantageous in terms of efficacy, formulation, and administration. Detailed analysis on the structure-activity relationships and the protective role of TMM-specific T cells against mycobacterial infection using primate models will contribute to the establishment of treatment and prevention options.

## Methods

### Sex as a biological variable

Our study examined human PBMCs from both male and female donors. Sex was not considered as a biological variable.

### Study participants

PBMCs from healthy donors were collected after obtaining informed consent. Peripheral and cord blood cells from healthy donors were also obtained from Veritas Corporation (Tokyo, Japan; batch 210570303C, 220771201C, 220771404C, 220772503C, 220781001C, 2208409001, 2208411000, 220873101C, 220880801C, 220881703C [PBMCs]; batch 2211410002, 2211416002, 2211422002, 2211422002, 2211422003, 2211423000, 2211423001, 2212406005, 2212414001, 2212414003, 2212420000 [cord blood mononuclear cells, CBMCs]). Active TB cases (13 cases) are those of patients who were admitted to the hospital as sputum smear– and culture-positive pulmonary TB cases consecutively included in the study from April 2023 to July 2023. All participants were enrolled after giving written informed consent. Blood was taken for active TB before starting treatment.

### Bacteria

*M. tuberculosis* strain H37Rv was provided by Ikuya Yano (Japan BCG Laboratory, Kiyose, Japan). For inactivation, the bacterium was heated at 65°C for 1 hour, followed by incubation at 60°C overnight. *M*. *bovis* BCG was purchased from the Japan BCG Laboratory.

### Lipid extraction and purification for stimulation

*M. tuberculosis* strain H37Rv lipids were extracted as previously described (48). Briefly, 10 mL chloroform/methanol (2:1, vol/vol) or acetone was added to 100 mg bacteria and sonicated at 40°C for 10 minutes. The organic phase was collected, dried, and dissolved in chloroform/methanol (2:1, vol/vol) for storage and aliquoting into various assays as a crude lipid. For lipid fractionation, crude lipids were separated by high-performance, thin-layer chromatography (Merck) followed by charring with copper (II) acetate-phosphoric acid. TMM was purified from *M*. *tuberculosis* H37Rv, *M*. *tuberculosis* CDC1551, *M*. *bovis* BCG, *M*. *intracellulare*, *M*. *smegmatis*, *Rhodococcus* *equi*, and *R*. *sp* 4306, TDM was purified from *M*. *tuberculosis* CDC1551, and GMM, MMM was purified from *Rhodococcus ruber*, and GroMM was purified from *M*. *bovis* BCG as previously described (13, 49–53). Briefly, the heat-killed bacteria were sonicated in chloroform/methanol (2:1, vol/vol) for 15 minutes on ice, and water was added (1:20 total volume). The organic layer was collected and evaporated completely. The crude lipids were separated by thin-layer chromatography (Merck), and fractions were extracted. MA was purified from *M*. *tuberculosis* H37Rv as described previously (48). Synthetic GMM was provided by Adriaan Minnaard (University of Groningen, Groningen, Netherlands) (54). For stimulation of cells, lipids dissolved in chloroform/methanol (2:1, vol/vol) were diluted in isopropanol, applied to 96-well plates at 20 μL/well, and air-dried prior to adding media.

### MALDI-TOF MS analysis

TMM was detected by MALDI-TOF MS with an UltrafleXtreme (Bruker Daltonics). In brief, purified lipid fractions and TMM standards were dissolved in chloroform/methanol (3:1, vol/vol) at a concentration of 1 mg/mL, and 1 μL sample was applied directly to the sample plate, followed by addition of 1 μL 2,5-dihydroxybenzoic acid (10 mg/mL in chloroform/methanol, 1:1, vol/vol) as a matrix. The samples were analyzed in the reflection mode with an accelerating voltage operating in a positive mode of 20 kV (13, 55).

### Chemical synthesis

Reactions were carried out under a nitrogen atmosphere unless otherwise noted and monitored by thin-layer chromatography using Merck Silica Gel 60 F254 plates. Flash chromatography was performed using flash silica gel 60N (spherical neutral, particle size 40–50 μm, Kanto Chemical). Nuclear magnetic resonance (NMR) spectra were recorded using a Bruker Avance III (500 MHz) device with a Prodigy (nitrogen-based) cryoprobe or a JNM-ECZL600R (600 MHz) device with a ROYAL HFX probe. Chemical shifts were reported in the scale relative to CHCl_3_ (δ 7.26 ppm for ^1^H NMR, 77.16 ppm for ^13^C NMR) or pyridine (δ 7.58 ppm for ^1^H NMR, 135.91 ppm for ^13^C NMR) as an internal reference. Splitting patterns are designated as s, singlet; d, doublet; t, triplet; q, quartet; br, broadening; and m, multiplet. High-resolution mass spectrometry (HRMS) was done with a Bruker MicrOTOF II detector or a Bruker MALDI-TOF MS Autoflex Speed device. Gel permeation chromatography (GPC) was executed using LaboACE LC–5060 equipped with JAIGEL-1HR and JAIGEL-2HR (CHCl_3_). HPLC purification was performed on the HITACHI HPLC system consisting of the following: pump, L6250; detector, L-3350 RI monitor; column, Senshu-Pak PEGASIL silica SP100; mobile phase, hexane/EtOAc.

#### TMM (C32, RR).

[α]_D_^27^ +110.01 (c = 0.28, CHCl_3_/MeOH 4/1); ^1^H NMR (500 MHz, CDCl_3_/CD_3_OD 4/1) δ 4.97 (d, *J* = 3.7 Hz, 1H), 4.93 (d, *J* = 3.7 Hz, 1H), 4.48 (dd, *J* = 11.9, 1.8 Hz, 1H), 4.05 (ddd, *J* = 10.0, 7.0, 1.8 Hz, 1H), 3.94 (dd, *J* = 11.9, 7.0 Hz, 1H), 3.75 (m, 1H), 3.72 (dd, *J* = 11.0, 1.8 Hz, 1H), 3.69 (dd, *J* = 9.8, 9.2 Hz, 1H), 3.67 (dd, *J* = 9.8, 9.2 Hz, 1H), 3.56–3.50 (m, 2H), 3.42 (dd, *J* = 9.8, 3.7 Hz, 1H), 3.37 (dd, *J* = 9.8, 3.7 Hz, 1H), 3.18 (dd, *J* = 9.6, 9.2 Hz, 1H), 3.14 (dd, *J* = 10.0, 9.2 Hz, 1H), 2.29 (ddd, *J* = 10.1, 7.8, 4.6 Hz, 1H), 1.50–1.39 (m, 2H), 1.38–1.28 (m, 2H), 1.28–1.05 (m, 50H), 0.75 (t, *J* = 7.0 Hz, 6H); ^13^C NMR (126 MHz, CDCl_3_/CD_3_OD 4/1) δ 175.5, 94.43, 94.37, 72.7, 72.62, 72.58, 72.3, 71.6, 71.4, 71.0, 70.8, 70.0, 64.0, 61.9, 52.5, 34.7, 31.8 (2C), 29.64–29.48 (15C), 29.4, 29.34, 29.26 (2C), 29.2, 27.2, 25.2, 22.6 (2C), 13.9 (2C); HRMS-MALDI (*m/z*): [M+Na]^+^ calcd for C_44_H_84_NaO_13_, 843.5810; found 843.58.

#### TMM (C32, SS).

[α]_D_^27^ +8.03 (c = 0.22, CHCl_3_/MeOH 4/1); ^1^H NMR (500 MHz, CDCl_3_/CD_3_OD 4/1) δ 4.98 (d, *J* = 3.7 Hz, 1H), 4.95 (d, *J* = 3.7 Hz, 1H), 4.29 (dd, *J* = 11.7, 2.0 Hz, 1H), 4.19 (dd, *J* = 12.1, 5.0 Hz, 1H), 3.92–3.87 (m, 1H), 3.72–3.66 (m, 4H), 3.57–3.50 (m, 2H), 3.41 (dd, *J* = 9.8, 3.7 Hz, 1H), 3.37 (dd, *J* = 9.8, 3.7 Hz, 1H), 3.24 (dd, *J* = 9.3, 9.2 Hz, 1H), 3.19 (dd, *J* = 9.3, 9.2 Hz, 1H), 2.29 (ddd, *J* = 10.2, 7.8, 4.6 Hz, 1H), 1.49–1.39 (m, 2H), 1.38–1.28 (m, 2H), 1.28–1.03 (m, 50H), 0.74 (t, *J* = 7.0 Hz, 6H); ^13^C NMR (126 MHz, CDCl_3_/CD_3_OD 4/1) δ 175.5, 94.0, 93.9, 72.8, 72.7, 72.5, 72.2, 71.6 (2C), 70.7, 70.3, 70.2, 63.1, 61.9, 52.9, 34.7, 31.8 (2C), 29.6–29.5 (15C), 29.4, 29.3, 29.2 (2C), 29.1, 27.2, 25.1, 22.6 (2C), 13.9 (2C); HRMS-MALDI (*m/z*) [M+Na]^+^ calcd for C_44_H_84_NaO_13_, 843.5810; found 843.58.

#### TMM (C32, RS +SR).

[α]_D_^27^ +9.02 (c = 0.23, CHCl_3_/MeOH 4/1); ^1^H NMR (500 MHz, CDCl_3_/CD_3_OD 4/1) δ 4.97 (d, *J* = 3.7 Hz, 1H), 4.96 (d, *J* = 3.4 Hz, 1H), 4.94 (d, *J* = 3.7 Hz, 1H), 4.92 (d, *J* = 3.7 Hz, 1H), 4.51 (dd, *J* = 11.8, 1.8 Hz, 1H), 4.23–4.17 (m, 2H), 4.00–3.88 (m, 3H), 3.74–3.63 (m, 10H), 3.540 (dd, *J* = 12.7, 6.7 Hz, 1H), 3.538 (dd, *J* = 11.9, 6.1 Hz, 1H), 3.404 (dd, *J* = 9.8, 3.7 Hz, 1H), 3.399 (dd, *J* = 9.8, 3.7 Hz, 1H), 3.37 (dd, *J* = 9.6, 3.7 Hz, 1H), 3.35 (dd, *J* = 9.8, 3.7 Hz, 1H), 3.25–3.12 (m, 4H), 2.40–2.32 (m, 2H), 1.60–1.50 (m, 2H), 1.38–1.28 (m, 6H), 1.28–1.03 (m, 100H), 0.74 (t, *J* = 6.9 Hz, 12H); ^13^C NMR (126 MHz, CDCl_3_/CD_3_OD 4/1) δ 175.04, 174.98, 94.3, 94.1 (2C), 94.0, 73.0, 72.8 (2C), 72.7, 72.4, 72.3, 72.2 (2C), 71.6 (3C), 71.5, 70.9, 70.8, 70.7, 70.3, 70.0, 69.9, 63.8, 63.3, 61.9 (2C), 51.6, 51.3, 33.8, 33.5, 31.8 (4C), 29.65–29.48 (30C), 29.46 (2C), 29.4 (2C), 29.2 (4C), 27.9, 27.8, 26.6, 26.1 (2C), 25.8, 22.6 (4C), 13.9 (4C); HRMS-MALDI (*m/z*) [M+Na]^+^ calcd for C_44_H_84_NaO_13_, 843.5810; found, 843.58.

Other synthetic precursors and TMM analogs were synthesized as described in the Supplemental Methods.

The stereoselective synthesis of TMM (C32, *RR*) was carried out by a modified method of Nishizawa et al. (56): [α]_D_ +28.8 (c 0.49, CHCl_3_/MeOH = 1:1); ^1^H NMR (500 MHz, CDCl_3_/CD_3_OD = 1:1) δ 5.10 (d, *J* = 4.0 Hz, 2H), 4.49 (dd, *J* = 12.0, 2.3 Hz, 1H), 4.19 (dd, *J* = 12.0, 5.7 Hz, 1H), 4.11–4.04 (ddd, *J* = 9.7, 5.7, 2.3 Hz, 1H), 3.85–3.76 (m, 4H), 3.72–3.66 (m, 2H), 3.55–3.46 (m, 2H), 3.38–3.30 (m, 2H), 2.48–2.40 (m, 1H), 2.21–1.94 (m, 2H), 1.68–1.15 (m, 52H), 0.89 (t, *J* = 6.9 Hz, 6H); ^13^C{^1^H} NMR (150 MHz, CDCl_3_/CD_3_OD = 1:1) δ 174.7, 93.4, 93.3, 72.6, 72.5, 71.8, 71.22, 71.17, 70.2, 70.1, 69.5, 62.8, 61.0, 52.2, 33.9, 31.2, 29.0, 28.94, 28.89, 28.86, 28.8, 28.7, 28.6, 28.2, 26.7, 24.7, 21.9, 21.6, 13.0; HRMS (ESI-TOF) *m/z*: [M + Na]^+^ calcd for C_44_H_84_NaO_13_, 843.5804; found, 843.5778.

### Antibodies

Human Fc block (Fc1) was purchased from BD Pharmingen. Anti–human CD3 (HIT3a), anti–human CD19 (SJ25C1), TotalSeq-C Hashtags (LNH-94; 2M2), anti–mouse CD3 (2C11, 17A2), anti–mouse CD69 (H1.2F3), and anti–rat CD2 (OX-34), anti–human CD1a (HI149), anti–human CD1b (SN13), anti–human CD1c (L161), anti–human CD1d (51.1), mouse IgG1 κ isotype control (MG1-45),and mouse IgG2b κ isotype control (MPC-11) antibodies were purchased from BioLegend.

### In vitro stimulation of PBMCs

Cryopreserved human PBMCs were thawed and labeled by CTV (Thermo Fisher Scientific) and then quenched and washed with RPMI 1640 medium (MilliporeSigma) supplemented with 5% human AB serum (Gemini Bio), penicillin (MilliporeSigma), streptomycin (MP Biomedicals), and 2-mercaptoethanol (Nacalai Tesque). CTV-labeled PBMCs (10^6^ cells) were stimulated in the same medium with plate-coated 3 μg *M. tuberculosis*–crude lipids, 3 μg synthetic GMM, or 3 μg heat-killed *M. tuberculosis* H37Rv for 10 days. Recombinant human IL-2 (1 ng/mL, PeproTech), human IL-7 (5 ng/mL, PeproTech), and human IL-15 (5 ng/mL, PeproTech) were added at days 2, 5, and 8. After staining with anti–human CD3 antibody, CTV^lo^CD3^+^ cells were sorted with an SH800 Cell Sorter (Sony Biotechnology) and used for scTCR- and RNA-Seq analyses.

### Single-cell–based transcriptome and TCR repertoire analysis

Single-cell transcriptome and TCR repertoire analyses were performed using the Chromium Controller (10x Genomics) according to the manufacturer’s instructions, as previously described (57). Libraries were sequenced on an Illumina NovaSeq 6000 in the paired-end mode. The raw reads were processed by Cell Ranger version 6.0.0-7.1.0 (10x Genomics). TCR repertoire analysis was conducted using Scirpy version 0.11.1, and gene expression–based clustering was determined using Scanpy 1.9.1. UMAP plots, heatmaps, volcano plots, and differential expression analyses were performed using Seurat R package version 5.0.1.

### Bulk TCR-Seq

PBMCs (3 × 10^5^) were lysed in QIAzol (QIAGEN). Full-length cDNA was then synthesized using SMARTer technology (Takara Bio), and the variable regions of the TCRα and TCRβ genes were amplified using TRAC-/TRBC-specific primers. After sequencing of the variable region amplicons, each pair of reads was assigned a clonotype [defined as TR(A/B)V and TR(A/B)J genes and CDR3] using MiXCR software (58).

### APCs

For the preparation of cytokine-differentiated human monocytes, CD14^+^ monocytes were sorted from freshly isolated human PBMCs using a magnetic cell sorting (MACS) cell separation column (Miltenyi Biotec), followed by cultured in RPMI 1640 supplemented with 10% FBS, nonessential amino acids, 10 ng/mL human GM-CSF, and 10 ng/mL human IL-4 for 7 days. Human CD1b was cloned into the retroviral vector pMX-IRES-human CD8 (59) using Phoenix packaging cells and PEI MAX (Polysciences). Supernatant containing retroviruses was used for infection into the mouse DC line DC2.4 (American Type Culture Collection [ATCC]).

### TCR reconstitution and stimulation

TCRα and -β chain cDNA sequences were synthesized with eBlock (Integrated DNA Technologies [IDT]) and cloned into retroviral vectors pMX-IRES-rat CD2. TCRα mutants were constructed by site-directed mutagenesis. The vectors were transduced into mouse T cell hybridoma with an NFAT-GFP reporter gene (57, 60) using retroviruses described above to reconstitute TCRαβ pairs. For antigen stimulation, TCR-reconstituted cells were cocultured with stimulants in the presence of APCs unless indicated otherwise. After 20 hours, T cell activation was assessed according to GFP and CD69 expression.

### NGS-based mutagenesis scanning

For the mutant libraries, synthesized mutant TCRα or β cDNA sequences were pooled, and reconstituted into reporter cell lines with WT TCRβ or TCRα, respectively. Library cells were left unstimulated or stimulated with TMM for 20 hours and then sorted by GFP^–^/GFP^+^ populations. Each sorted cell population was analyzed by bulk TCR-Seq (GEO GSE261269). The proportion of the read counts of each mutant within the GFP^+^ or GFP^–^ cell population are shown as a percentage.

### CD1b tetramers

Unloaded human CD1b monomers (biotinylated) were obtained from the NIH tetramer facility. For TMM loading, 16 μg *M. tuberculosis* TMM was sonicated at 45°C for 1 hour in 45 μL 0.5% CHAPS 50 mM sodium citrate buffer (pH 4.5), added to 5 μL CD1b monomers (2 mg/mL) and incubated overnight at 37°C. For the preparation of control CD1b tetramers (endo-CD1b tet), CD1b monomers were treated as described above without TMM loading. Monomers were then neutralized by 5 μL 1 M Tris (pH 8) and tetramerized using streptavidin-PE (BioLegend) or streptavidin-APC (eBioscience).

### Tetramer staining and isolation of CD1b tetramer+ T cells

Human PBMCs (107) were incubated with 20 μg/mL PE-conjugated TMM-CD1b tetramers and 20 μg/mL APC-conjugated endo-CD1b tetramers in 40 μL 1% BSA/PBS at room temperature in the dark for 15 minutes. Without washing, 2 μL human Fc block (50 μg/mL), 60 μL anti–human CD3-FITC (2 μg/mL), and 2 μL TotalSeq-C Hashtags were added and incubated on ice for 20 minutes. Before sorting, cells were washed and filtered with a nylon mesh and incubated with propidium iodide. 2492 TMM-tetramer^+^ endo-tetramer^-^ cells within the CD3^+^ gated population were sorted with a SH800 Cell Sorter (Sony Biotechnology) and subjected to scTCR-RNA-Seq. Unsorted PBMCs from the same donors were also subjected to scTCR-RNA-Seq. Among the 1,737 TMM-tetramer^+^ cells (1,559 clonotypes) obtained, clonotypes that were detected more abundantly in unsorted T cells than in TMM-tetramer^+^ T cells were excluded.

### Characterization of Y-50 before and after TMM stimulation

For unstimulated cells, PBMCs from 3 donors were stained with anti-CD3Ab, and PE-conjugated TMM-CD1b tetramer and CD3^+^TMM-tetramer^+^ cells were sorted. For TMM-simulated cells, CTV-labeled PBMCs from 5 donors were stimulated with TMM for 8–10 days, and CD3^+^CTV^lo^ cells were sorted. These samples were subjected to scTCR-RNA-Seq analysis and projected on the same UMAP plot. On the basis of scTCR-Seq, the cells expressing a clonotype identical to Y-50 TCRαβ were designated as a Y-50 clonotype.

### Crystal structural analysis

cDNA encoding the ectodomains of Y-50 TCRα (from Ala-1 to Ser-205) and β (from Asp-1 to Asp-249) with a nidogen signal sequence, a 6 × His-tag, and a tobacco etch virus protease cleavage site at the N-terminal were synthesized (Thermo GeneArt) and subcloned into a pcDNA3.1(+) vector. To improve the efficiency of protein expression, artificial disulfide bond and stabilizing mutations were introduced as described previously (61, 62). The plasmids were transformed into Expi293 cells in the presence of the mannosidase inhibitor kifunensine. The cells were then cultured with shaking at 120 rpm 37°C 8% CO_2_ for 4 days. After being passed through a 0.22 μm filter, the supernatant was applied to 5 mL nickel-nitrilotriacetic acid agarose (FUJIFILM Wako), and His-tagged TCRαβ were eluted with elution buffer (50 mM Tris-HCl [pH 8.0], 300 mM NaCl, and 250 mM imidazole). After removal of the His-tag by tobacco etch virus protease, the eluted protein was concentrated and further applied to Superdex 75 (Cytiva) equilibrated with 20 mM Tris-HCl (pH 8.0) buffer containing 100 mM NaCl. The crystals were formed by the sitting-drop, vapor-diffusion method. A 0.4 μL protein solution (5 mg/mL in 100 mM NaCl, 20 mM Tis-HCl [pH8.0]) was mixed with 0.4 μL mother liquid containing 0.2 M potassium sulfate 0.1 M Bis-Tris (pH 5.5) and 25% PEG3350 and incubated at 20°C. The diffraction data were collected in a cold nitrogen gas stream on an EIGER X 9M detector (DECTRIS) at a wavelength of 1.0 Å. The resulting datasets were processed, integrated by XDS (63), and scaled by AIMLESS (64). Structures were clarified by molecular replacement with the TCR complex (PDB: 8ZO4 as a search model, by MOLREP) as implemented in CCP4i software (64). The models were refined using REFMAC5 and PHENIX1.20 software (65, 66). The structures were rebuilt using COOT 0.9.8.92 (67) and further modified based on σ-weighted (2|*F*_obs_|—|*F*_calc_|) and (|*F*_obs_|—|*F*_calc_|) electron density maps. Crystallographic images were created using PyMOL software (Schrödinger). Data collection and refinement statistics are summarized in Supplemental Table 2.

### Cryo-EM structural analysis

The TMM-loaded CD1b ectodomain was refolded as follows: denatured proteins of CD1b (24.8 mg) and β2m (9.6 mg) were mixed with 3.28 mg TMM and refolded in the buffer containing 0.1 M Tris-HCl (pH 8.0), 1 M l-arginine (pH 8.0), 5 M urea, 5 mM reduced glutathione, and 0.5 mM oxidized glutathione. The refolded proteins were then dialyzed 4 times against 0.01 M Tris-HCl (pH 8.0) and applied onto a HiTrap Q HP 5 mL column (Cytiva). Purified TMM-loaded CD1b was mixed with Y-50 TCR at a 1:1 ratio. A 2.2 μL sample (1.0 mg/mL) was applied onto the glow-discharged Quantifoil Au 0.6/1.0 200 mesh grid (Quantifoil Micro Tools) and frozen in liquid ethane using a Vitrobot IV (FEI, 4°C and 95% humidity). Cryo-EM data collection was performed on a Titan Krios cryo-TEM equipped with a Cs corrector (Thermo Fisher Scientific, USA) operating at 300 keV in EFTEM nanoprobe mode. Images were acquired as movies using a Gatan BioQuantum energy filter (slit width of 20 eV) and a K3 direct electron detector camera (Gatan) in electron counting mode. A total of 8,533 movies were collected at a dose rate of 8.532 e^–^/pixel/s, a pixel size of 0.675 Å^2^, and a total dose of 60 e^–^/Å^2^. SerialEM software (68) was used for automated data collection using a 3 ′ 3-hole pattern beam-image shift scheme with a nominal defocus range of –0.6 to –1.8 μm. All image processing was carried out using cryoSPARC version 4.4.1 software (69). After motion correction of movies and contrast transfer function (CTF) parameter estimation, an initial round of particle picking was performed using the blob picker tool (diameter 100–140 Å). After 4 iterations of 2D class and manual selection, 12,424 particles were selected. Classification into 3 classes using Ab Initio reconstruction and manual selection was repeated twice, and the resulting 6,961 particles were used as training data for Topaz picks. The 2,266,363 particles were automatically picked using the Topaz picking algorithm. After 2 rounds of 2D classification and 3D classification using Ab Initio Reconstruction and Heterogeneous refinement, 599,402 particles were selected. A subsequent round of 2D classification further narrowed the selection to 232,909 particles. Three maps were reconstructed using Ab-initio reconstruction with C1 symmetry. Several maps were duplicated and used as the initial model for the heterogeneous refinement. In this process 2,266,363 particles picked by TOPAZ were used, but the resolution was not high enough, so 3,158,103 particles were picked by the blob picker. As a result, one of these classified particles (599,402 particles) were applied to 2D classification, Ab-initio reconstruction, and nonuniform refinement. Finally, 11,404 particles were selected, and the density map from the refinement was obtained at 3.31 Å resolution. Each particle was subjected to reference-based motion correction. The results of nonuniform refinement were produced in a map of the complex at 3.18 Å resolution. Local resolution of the obtained map was estimated by a local resolution estimation job on cryoSPARC. 3D structures of Y-50 (PDB: 8XUB) and CD1b (PDB: 5L2K) were automatically fitted into the map with program phenix.dock_in_map in the PHENIX program suite (66). Chemical structure of TMM was idealized by phenix.elbow. The atomic model of ternary complex was manually modified using COOT and refined with the phenix.real_space_refine of PHENIX suite. Stereochemistry of the refined structure was evaluated with MolProbity (70). Validation of the final model is summarized in Supplemental Table 3.

### Statistics

Data were analyzed with GraphPad Prism version 9.1.0 software (GraphPad Software). Statistical differences between 2 groups were determined by unpaired 2-tailed Welch’s *t* test. A *P* value of less than 0.05 was considered statistically significant. Data are presented as the mean ± SD.

### Study approval

The protocol for collecting human blood samples from healthy donors was approved by the IRB of Osaka University (approval no. 898-4). Informed consent was obtained from all participants before the first blood sampling. The protocol for collecting human blood samples from patients with active TB was reviewed and approved by the medical research ethics committee of the National Institute of Infectious Diseases for inclusion of Human Subjects (nos. 1343 and 1491) and Fukujuji Hospital (no. 22034). All participants were enrolled after giving written informed consent.

### Data availability

All reagents used in this study will be made available upon reasonable request to the corresponding author. All single-cell TCR-RNA-Seq and Bulk TCR-Seq data were deposited in the NCBI GEO database (GSE260931 and GSE261269). Values for all data points in graphs are provided in the Supporting Data Values file.

## Author contributions

YS, MA, MH, WK, XL, and EI performed studies. NF, HT, TI, SO, KK, TY, YH, IVR, SI, and GH provided resources. MH, KT, MN, DM, and TK handled data curation. SY supervised the research. YS, MA, DBM, and SY wrote the manuscript.

## Supplementary Material

Supplemental data

Unedited blot and gel images

Supplemental video 1

Supplemental video 2

Supplemental video 3

Supporting data values

## Version 1 12/24/2024

In-Press Preview

## Version 2 03/17/2025

Electronic publication
