# Supplementary material for: A conserved human CD4+ T cell subset recognizing the mycobacterial adjuvant trehalose monomycolate
Source: J Clin Invest. 2024 Dec 24;135(6):e185443. doi: 10.1172/JCI185443 (PMC11910211; doi:10.1172/JCI185443)
Supplement: Supplemental data [file jci-135-185443-s251.pdf]

Supplemental Figure 1

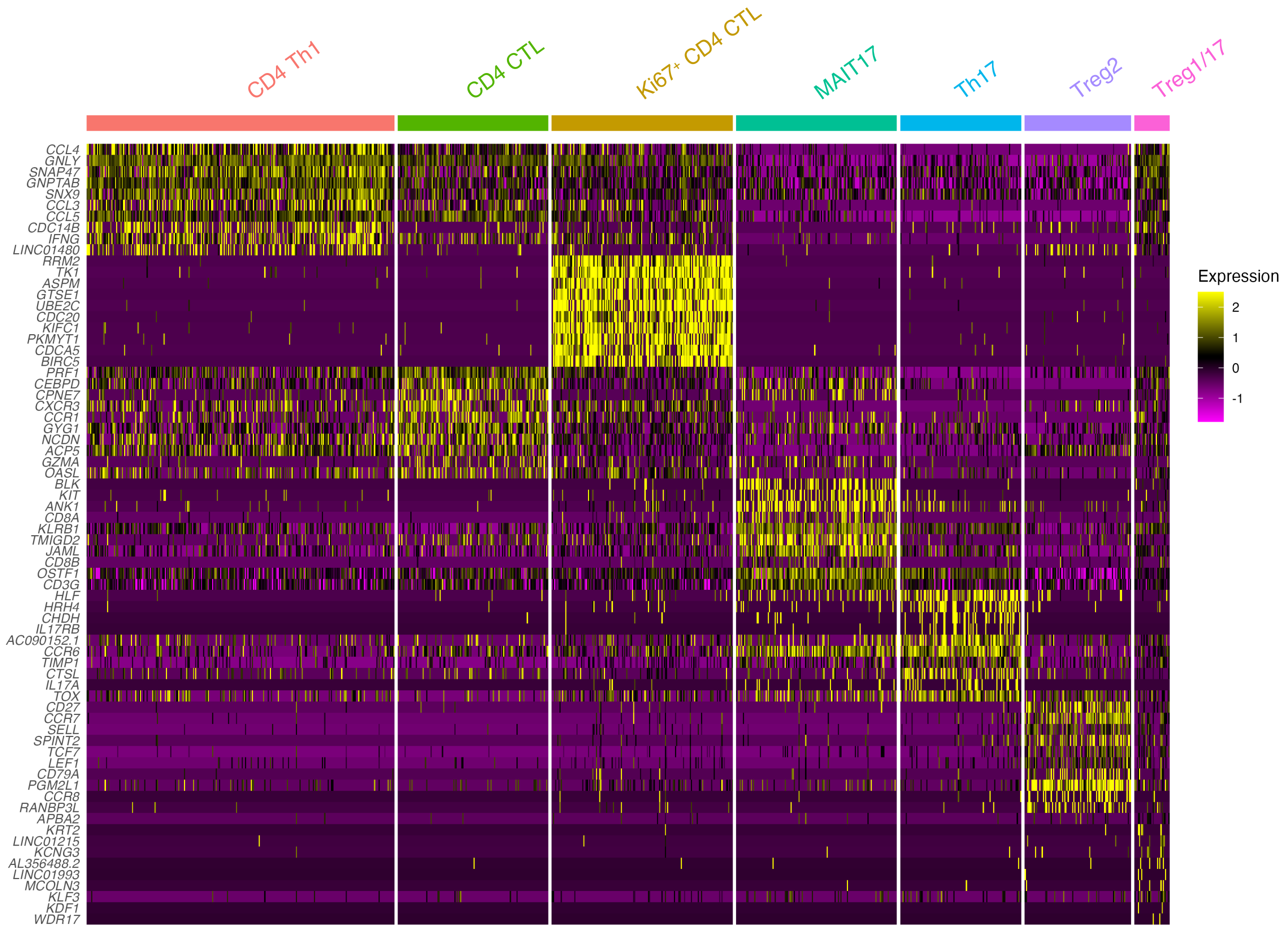

Supplemental Figure 1. Characteristic genes in each cluster of UMAP plot in Figure 1D.
Log-normalized expression of the top 10 genes for each T cell cluster in Figure 1D are shown in heatmap. The colors of annotated clusters are consistent with the dots shown in Figure 1D.

Supplemental Figure 2

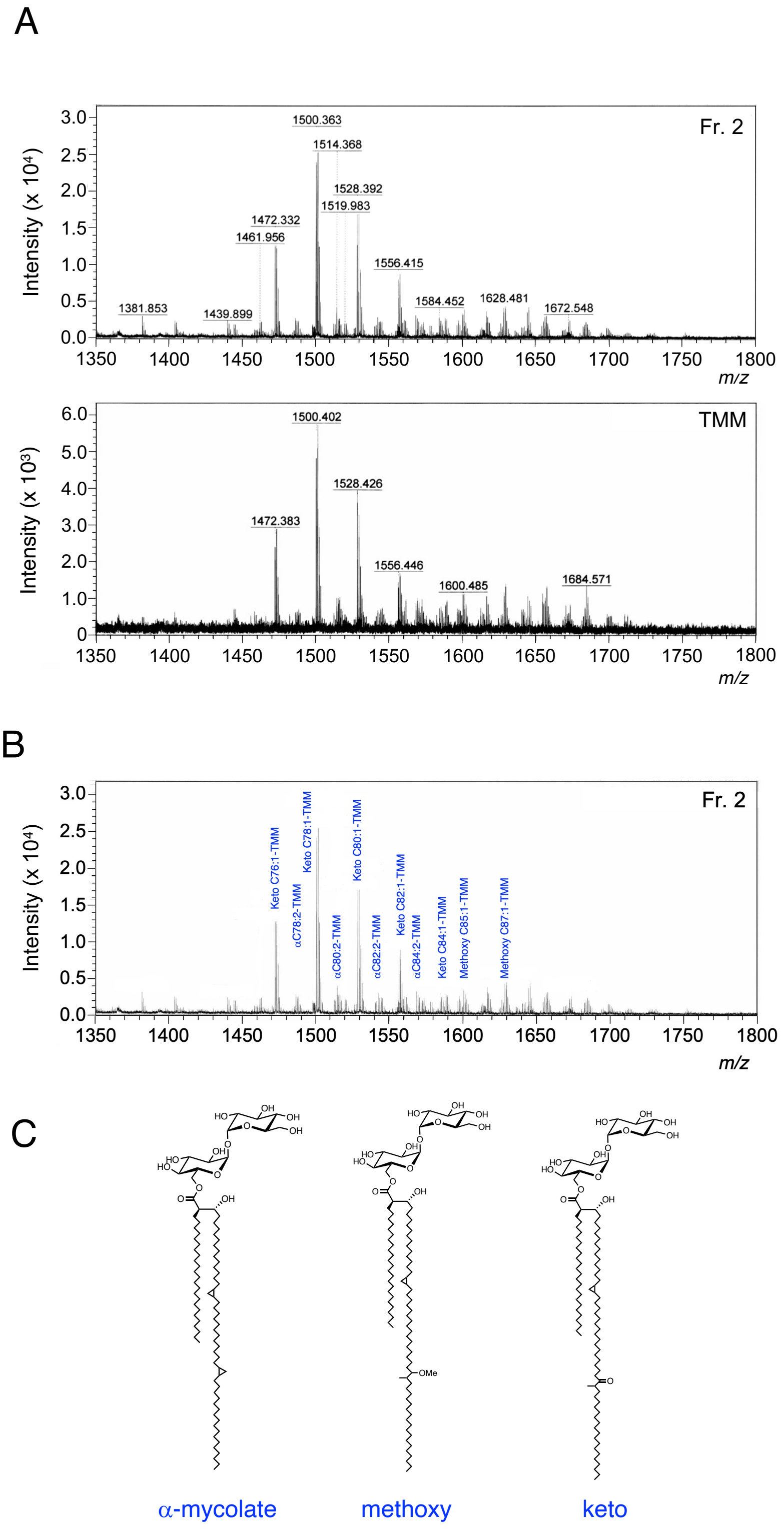

Supplemental Figure 2. Identification of antigen recognized by Y-50.
A-B) MALDI-TOF MS spectrum of lipid fraction 2 (Fr2) and the reference spectrum of *M. tuberculosis* H37Rv TMM (lower) (A). The subclass of mycolate and chain length annotated based on the detected *m/z* matching the structural formula were indicated on upper panel of (A) (B). Related to Figure 2C.
C) Structural characteristics of TMM composed of three mycolic acid subclasses.

Supplemental Figure 3

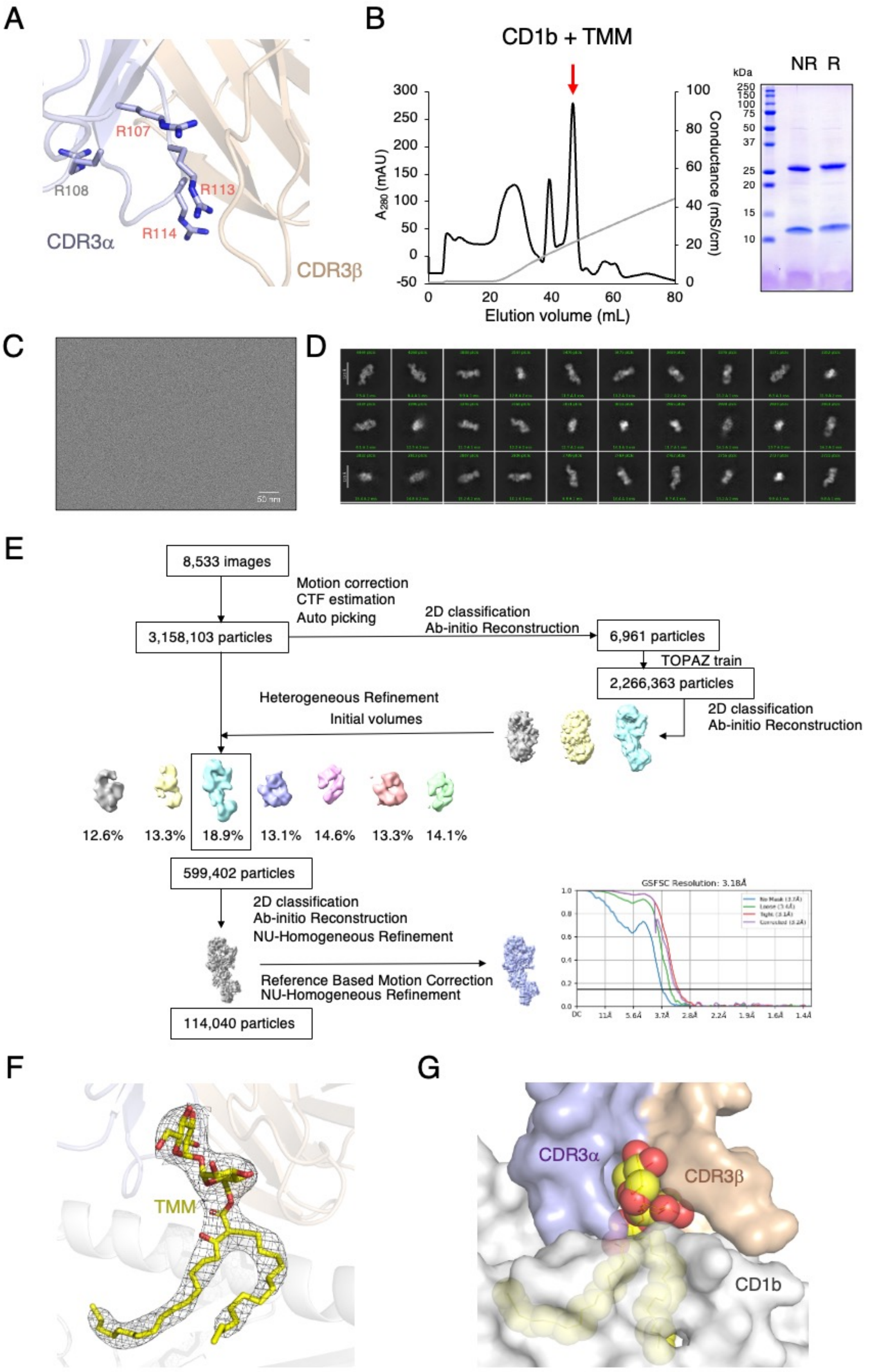

Supplemental Figure 3. Structural analysis of Y-50 TCR-TMM-CD1b ternary complex.
A) Close-up view of the side chains of CDR3α arginine residues (R107, R108, R113 and R114) in the crystal structure of Y-50 (PDB 8XUB).
B) Refolding of recombinant CD1b (CD1b) with synthetic TMM (TMM). Red arrow indicates refolded CD1b-TMM-β2m complex separated by anion-exchange chromatography (left), which was confirmed by SDS-PAGE followed by CBB staining (right). NR, non-reducing; R, reducing.
C-D) Representative micrograph (C) and 2D classification (D) of cryo-EM analysis.
E) Flow chart of cryo-EM image processing. Gold standard FSC curves are also shown.
F) Cryo-EM density map (gray mesh) and a model structure TMM (yellow).
G) TMM (yellow sphere) and surface representation of the CDR3 (violet), CDR3β (brown) loops and CD1b (gray) are shown. Oxygen atoms of TMM are shown in red.

Supplemental Figure 4

A

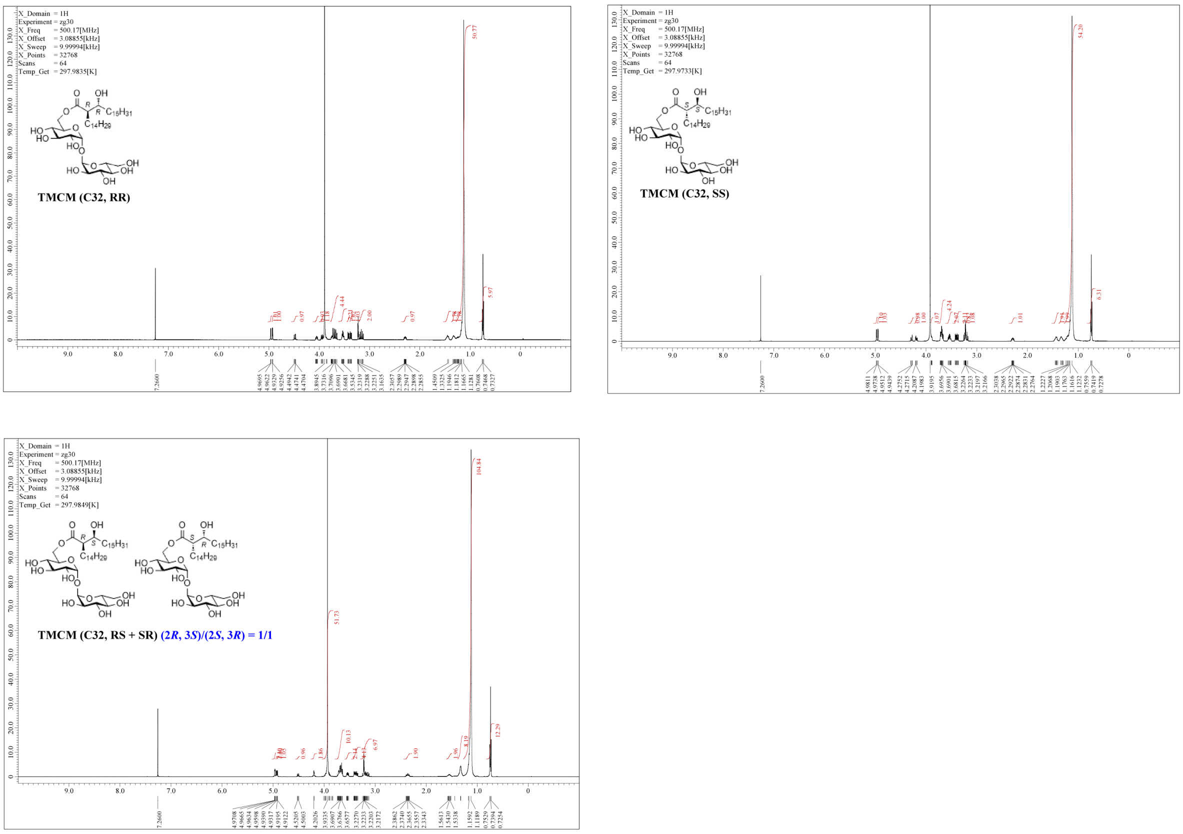

B

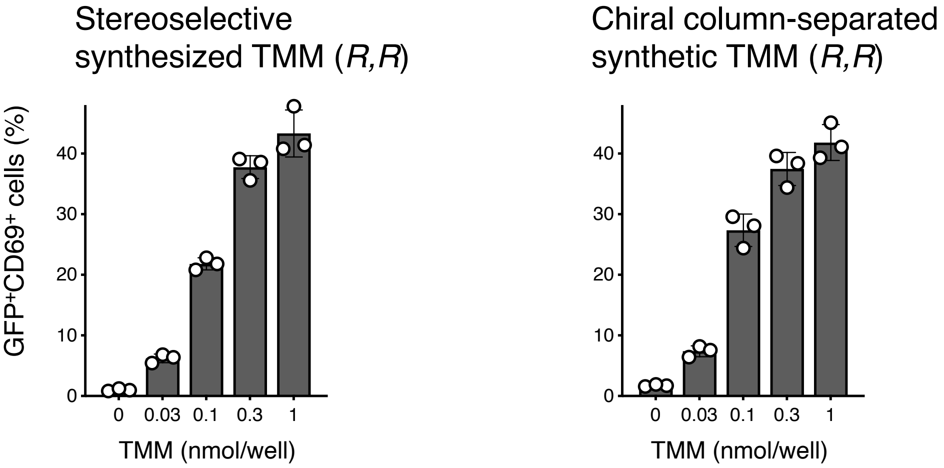

Supplemental Figure 4. Synthesis of TMM.

A) NMR chart of synthetic TMMs ((*R*,*R*), (*S*,*S*) and (*R*,*S* + *S*,*R*)).

B) Antigenic activity of synthetic TMM. Y-50 reporter cells were stimulated with stereoselective synthesized (left) or chiral column-separated synthetic (right) TMM (*R*,*R*) in the presence of CD1b-DC2.4 and analyzed for GFP and CD69 expression. Data are shown as the means ± SD of triplicate assays and representative results from two independent experiments.

Supplemental Figure 5

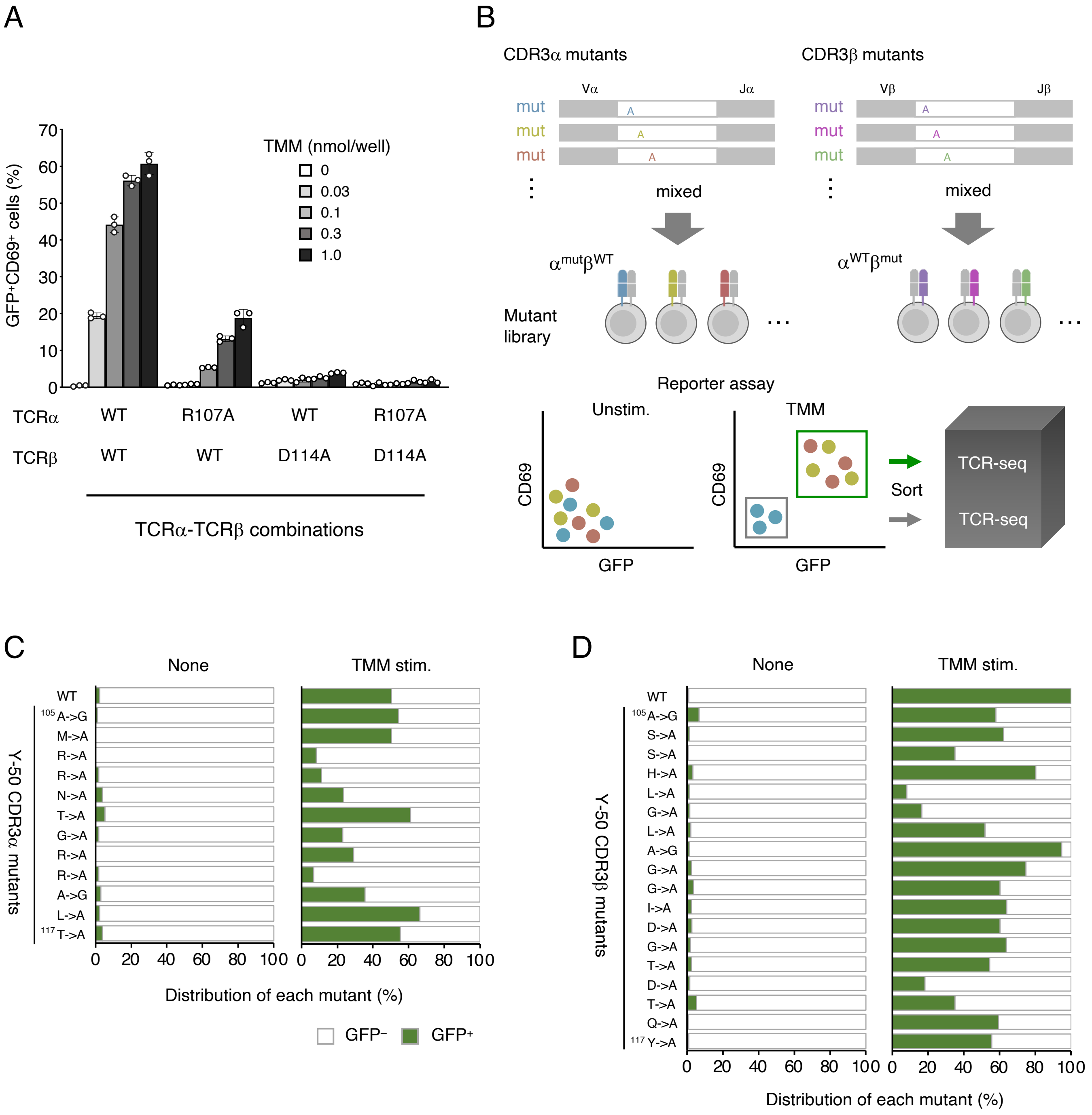

Supplemental Figure 5. Mutagenesis analysis of Y-50 TCR.

A) Reporter cells expressing indicated combination of Y-50 TCRαβ mutants were stimulated with TMM on CD1b-DC2.4 and percentages of $GFP^{+}CD69^{+}$ cells are shown. Data are shown as the means ± SD of triplicates and a representative result from two independent experiments is shown.

B) Schematic procedure of NGS-based mutagenesis scanning.

C-D) Distribution of reporter cells expressing each mutant in GFP^{+} and GFP^{-} populations with or without TMM stimulation. The ratio of each CDR3α mutant (B) and CDR3β mutant (C) are shown as percentages. The number of amino acids were shown in accordance with the ImMunoGeneTics (IMGT) definition.

Supplemental Figure 6

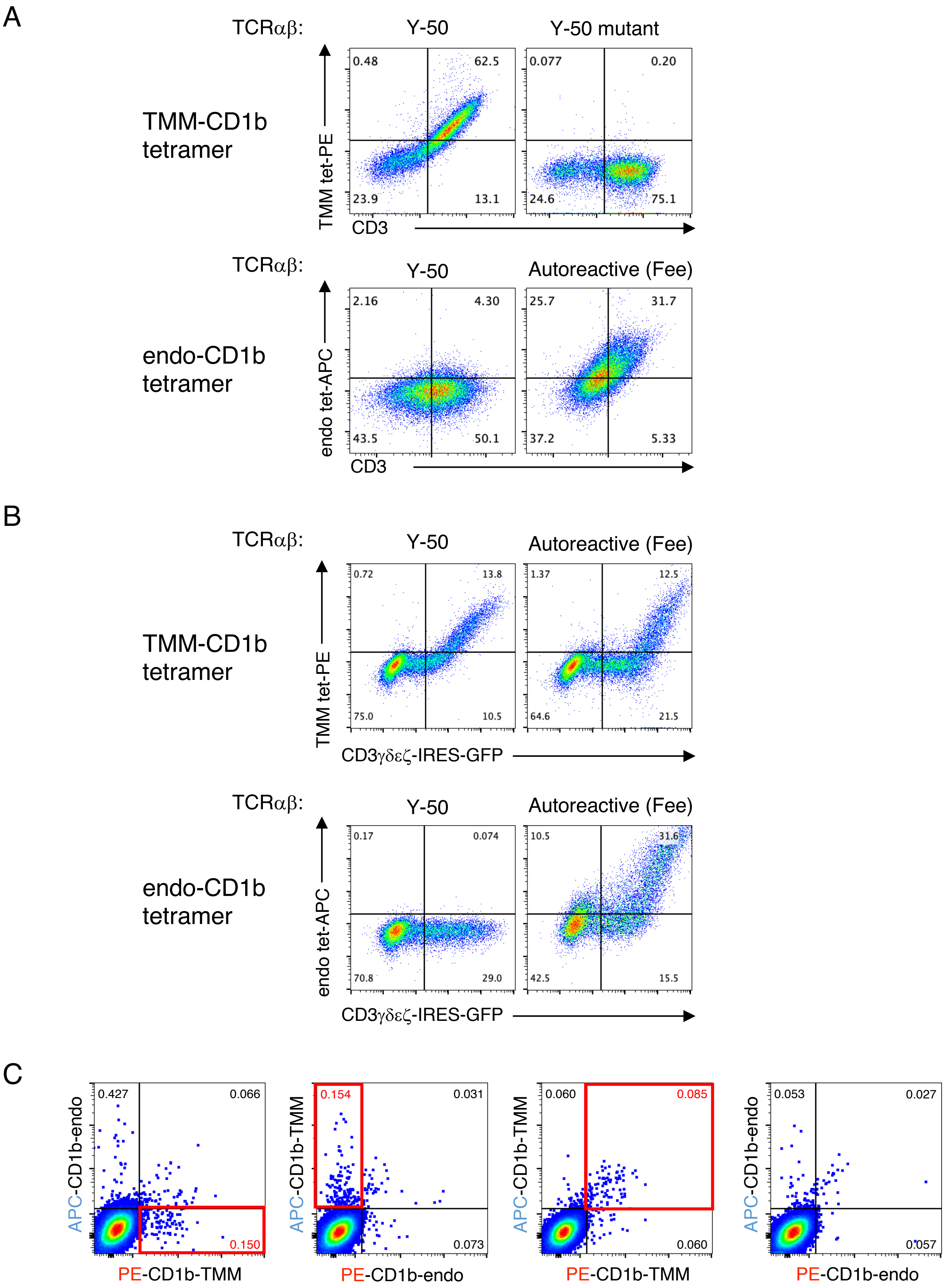

Supplemental Figure 6. Validation of TMM-loaded CD1b tetramers.

A) Reporter cells expressing Y-50 and mutant Y-50 TCR shown in Figure 3B (Δ12) were stained with PE-conjugated TMM-loaded CD1b tetramer and anti-CD3 antibody. As a control tetramer, CD1b biotinylated monomer conjugated by SA-APC (endo-CD1b tet) was prepared. Endo-CD1b tet reactivity was confirmed by Y-50 (negative control) and autoreactive Fee TCR (Gherardin et al., 2021) (positive control).

B) HEK 293T cells were transfected with plasmids encoding Fee TCR and human CD3s-IRES-GFP and stained with PE-conjugated TMM-CD1b or APC-conjugated endo-CD1b tetramers.

C) TMM-stimulated PBMCs were co-stained with all combination of four tetramers (APC-labeled CD1-TMM, PE-labeled CD1-endo tetramers, PE-labeled CD1-TMM and APC-labeled CD1-endo tetramers). Numbers in red indicate the frequency of TMM-specific T cells with $CD3^+$ cells for each staining combination.

Supplemental Figure 7

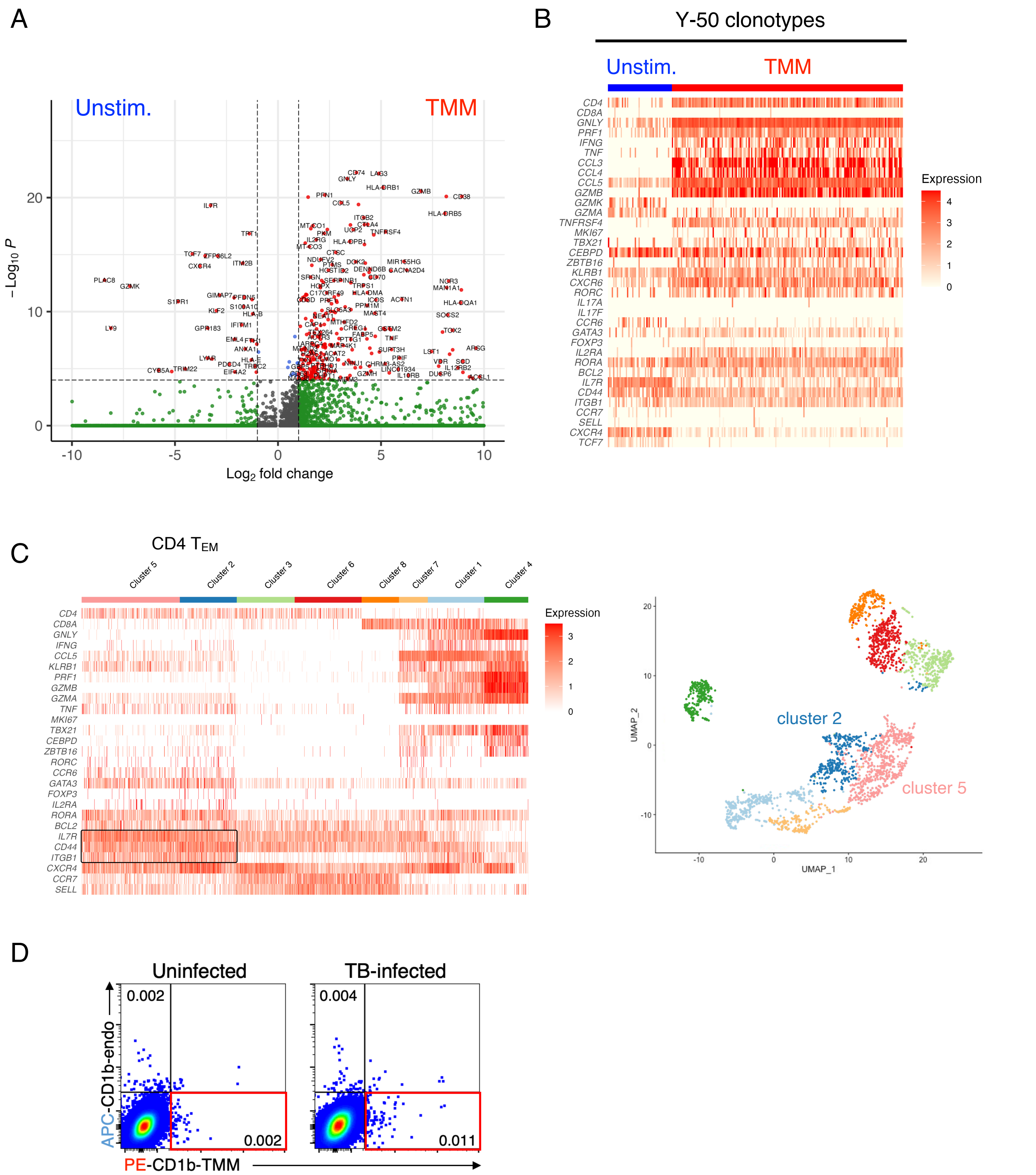

Supplemental Figure 7. Gene expression profile of TMM-specific T cells with or without TMM stimulation.

A) Volcano plot showing differentially expressed genes of Y-50 T cells in response to TMM stimulation.

B) Heatmap showing log-normalized expression of marker genes in individual Y-50 cells in the presence or absence of TMM stimulation.

C) Heatmap showing log-normalized expression of marker genes for each CD3$^+$ T clusters shown in Figure 7A-B. Typical signature of CD4$^+$ effector memory T cells is boxed.

D) Representative plots of the tetramer staining of PBMCs from uninfected (left) and TB-infected (right) donors. PBMCs were stained with PE-conjugated TMM-loaded CD1b tetramer, APC-conjugated CD1b-endo tetramer and anti-CD3 antibody. Numbers within boxes indicate the percentages of TMM-CD1b tetramer positive and endo-CD1b tetramer negative population in CD3$^+$ T cells are shown.

Supplemental Table 1. Mycobacterial lipid-reactive candidate T cells in human PBMCs.

Ex#	clone ID	TRAV	CDR3α	TRAJ	TRBV	CDR3β	TRBJ
U	2	1-2	CAAMDSNYQLIW	33	6-4	CASSDGGGTDTQYF	2-3
	14	8-3	CAVGGGGYQKVTF	13	11-2	CASSLVSSGALTEQYF	2-7
	15	1-2	CAVVDSNYQLIW	33	6-5	CASSPGSGVAEQYF	2-7
	53	20	CAVRVYGQNFVF	26	20-1	CSALQPGLAGPQITYEQYF	2-7
	59	1-2	CAVRDSNYQLIW	33	6-1	CASSPSTGGSSPLHF	1-6
	86	1-2	CAVRDGDYKLSF	20	4-3	CASSQDGSSGANVLTF	2-6
	104	1-2	CAVRDSNYQLIW	33	4-3	CASSLATEPSYEQYF	2-7
	221	1-2	CAAMDSNYQLIW	33	20-1	CSAKGPAGADTGELFF	2-2
V	1	1-2	CAVVDSNYQLIW	33	6-1	CASSSSGSAADTQYF	2-3
	2	27	CAGGNSGNTPLVF	29	4-1	CASRREGENIQYF	2-4
	3	8-6	CAVSDGRRDDKIIF	30	7-6	CASSPLQRGTYEQYF	2-7
	153	29/DV5	CAASTYGGATNKLIF	32	30	CAWSRRLQVLNGYTF	1-2
	569	5	CAEIPYTGGGNKLTF	10	4-1	CASSQGGGATGNTIYF	1-3
	570	8-4	CAVSVGLGFGNVLHC	35	6-5	CASSYSPGVWPQHF	1-5
	573	8-1	CAVNAPHGEKLVF	8	5-1	CASSPGTSGVYNEQFF	2-1
	597	19	CALSERSTGNQFYF	49	6-5	CASSYSPGVWPQHF	1-5
	606	17	CATVPNNDYKLSF	20	6-1	CASTYSTMNTEAFF	1-1
	648	1-2	CAVKDNYGQNFVF	26	30	CAHRTRGETQYF	2-5
	828	41	CAAFGNEKLTF	48	30	CAWSVSSGWPLHF	1-6
	835	17	CATDRAGANNLFF	36	4-1	CASSQDPGNEKLFF	1-4
	1030	14/DV4	CAMPAGTGRRALTF	5	11-3	CASSLTSGGYGGTDTQYF	2-3
	1176	1-2	CAVKDSNYQLIW	33	4-2	CASSVERGAGANVLTF	2-6
X	23	12-2	CAANSGGSNYKLTF	53	30	CAWTRETGQPQHF	1-5
	34	23/DV6	CAALHGSSNTGKLIF	37	11-2	CASSDWTSSSGSYEQYF	2-7
	84	8-4	CAVRPAAGNKLTF	17	28	CASMADYNEQFF	2-1
	291	3	CAVRVLSGGYNKLIF	4	14	CASSRRPYSGSTDTQYF	2-3
	309	29/DV5	CAASAKTSGSRLTF	58	7-2	CASSLDPGLAKNIQYF	2-4
	605	5	CAESIWGSQGNLIF	42	19	CASSIDGRQLGSFF	1-1
	700	1-2	CAVSALYGQNFVF	26	11-2	CASSDWTSSSGSYEQYF	2-7
Y	0	8-2	CVVRLYQKVTF	13	14	CASSQGYRGSSYNEQFF	2-1
	5	2	CAVPSNTGKLIF	37	7-8	CASSLAQGTGNNSPLHF	1-6
	10	38-1	CAFTPNTGNQFYF	49	4-1	CASSQEFRDTEAFF	1-1
	11	5	CAESIGTGGFKTIF	9	30	CAWSVLAGRGETQYF	2-5
	13	12-2	CAVNGGGFKTIF	9	7-3	CASSLTPKRSNTGELFF	2-2
	14	14/DV4	CAMREGMGGNMLTF	39	20-1	CSARHSASSYEQYF	2-7
	16	12-3	CAKGFNDYKLSF	20	7-8	CASSLRGHLQETQYF	2-5
	17	17	CATLRNTNAGKSTF	27	20-1	CSAVLTDTQYF	2-3
	18	14/DV4	CAMRGSNTGKLIF	37	7-9	CASSLAGGQGLAYTF	1-2
	22	8-3	CAVGNSGGYQKVTF	13	7-2	CASSPGAYEQYF	2-7
	24	17	CATKVTSGGSYIPTF	6	20-1	CSALTSGRPDTQYF	2-3
	28	9-2	CALSETDSWGKLQF	24	20-1	CSARGLAGGQYF	2-7
	29	2	CAVERDFNKFYF	21	20-1	CSAPLAGGQFF	2-1
	36	1-2	CAVMDSNYQLIW	33	6-1	CASSDRGHSPLHF	1-6
	50	14/DV4	CAMRRNTGRRALTF	5	4-1	CASSHLGLAGGIDGTDTQYF	2-3
	56	8-4	CAVSPMIYNQGGKLIF	23	14	CASSQRGQVKGNTIYF	1-3
	62	14/DV4	CAILYNFNKFYF	21	12-4	CASSLAQIAKNIQYF	2-4
	66	20	CAVQARTQGGSEKLVF	57	12-3	CASRDYLSTDTQYF	2-3
	83	22	CAVVLWGGADGLTF	45	10-1	CASSDGREQYF	2-7
	87	12-2	CAVSGSSNTGKLIF	37	4-2	CASSQEPQQETQYF	2-5
	92	1-2	CAVRDRDYKLSF	20	6-2	CASSYSDFNEQFF	2-1
	114	17	CATAGRIGARLMF	31	28	CASRPTSGRAYGETQYF	2-5
	151	8-6	CAVSTFSSGSARQLTF	22	11-2	CASSLASGRPTDTQYF	2-3

Supplemental Table 2. Data collection and refinement statistics of the crystallographic analysis of Y-50.

**Crystal name**	**Y-50**
**PDB ID**	**8XUB**
**Data collection statistics**	
Space group	$\boldsymbol{P\,2\,2_1\,2_1}$
Cell constants (Å)	a = 68.1 b = 168.7 c = 173.1
Resolution (Å)[a]	44.03 - 2.50 (2.59 - 2.50)
R-merge (%)[a]	2.62(42.8)
Completeness (%)[a]	99.9 (100.0)
$\langle I/\sigma I\rangle$[a]	17.1 (1.6)
Multiplicity[a]	2.0 (2.0)
**Refinement statistics**	
R (%)	*22.6*
R_{free} (%)	*29.9*
Root mean square deviations from ideal values	
Bond length (Å)	0.009
Bond angle (°)	1.07
Ramachandran plot	
Favored (%)	94.5
Outlier (%)	0
Rotamer outlier (%)	2.84

Statistics for the highest-resolution shell are shown in parentheses.

Supplemental Table 3. Cryo-EM data collection, refinement and validation statistics.

**PDB ID**	**8ZOX**
**EMDB ID**	**EMD-60321**
**Data collection and processing**	
Magnification	103,703
Voltage (kV)	300
Electron exposure (e^{-}/Å^2)	60
Defocus range (μm)	-0.6 to -1.8
Pixel size (Å)	0.675
Symmetry imposed	*C*1
Initial particle image	4,075,153
Final particle image	114,111
Map resolution (Å)	3.18
FSC threshold	0.143
Map sharpening *B* factor (Å^2)	118.4
**Refinement**	
Model composition	
Non-hydrogen atoms	6,590
Protein residues	813
Ligands	5
B factor (Å^2)	
Protein	125.4
Ligand	114.0
Root-mean-square deviations	
Bond lengths (Å)	0.005
Bond angles (°)	0.604
Validation	
MolProbity score	1.91
Clashscore	11.9
Poor rotamers (%)	0.71
Ramachandran plot	
Favored (%)	95.4
Allowed (%)	4.5
Disallowed (%)	0.1

Supplemental Table 4. Donor information.

Donor ID	Age (years)	Gender	Definition
F507	26	M	Active
F508	49	M	Active
F509	83	F	Active
F510	55	M	Active
F511	64	M	Active
F514	79	M	Active
F515	39	M	Active
F516	60	M	Active
F517	90	F	Active
F518	34	F	Active
F519	19	M	Active
F520	78	M	Active
F521	63	M	Active
MI001	27	M	Uninfected
MI002	25	M	Uninfected
MI003	24	F	Uninfected
MI004	27	M	Uninfected
MI011	60	F	Uninfected
MI012	27	F	Uninfected
MI013	36	F	Uninfected
NA001	49	M	Uninfected
NA002	31	M	Uninfected
NA003	44	M	Uninfected
NA004	22	F	Uninfected
NA005	33	F	Uninfected
NA006	28	M	Uninfected
NA007	27	M	Uninfected
NA008	22	F	Uninfected
NA009	40	F	Uninfected
NA010	56	F	Uninfected

Active: 56.8 + 22.6 y.o., male = 77%
Uninfected (JPN): 32.3 + 12.8 y.o., male = 23%
Uninfected (US): 35.2 + 11.6 y.o., male = 38%

Supplemental information

Extended Methods

Chemical synthesis

Experiments and Reagents

Reactions were carried out under a nitrogen atmosphere unless noted otherwise. Reactions were monitored by thin-layer chromatography using Merck Silica Gel 60 F254 plates. Flash chromatography was performed using flash silica gel 60N (spherical neutral, particle size 40–50 μm) purchased from Kanto Chemical Co. Ltd.

Instrumentation

NMR spectra were recorded Bruker Avance III (500 MHz) with a Prodigy (nitrogen-based) cryoprobe or JNM-ECZL600R (600 MHz) with ROYAL HFX probe. Chemical shifts were reported in the scale relative to CHCl3 (δ 7.26 ppm for 1H NMR, 77.16 ppm for 13C NMR) or pyridine (δ 7.58 ppm for 1H NMR, 135.91 ppm for 13C NMR) as an internal reference. Splitting patterns are designated as s: singlet, d: doublet, t: triplet, q: quartet, br: broadening, and m: multiplet. High-resolution mass spectrometry (HRMS) was obtained with Bruker MicrOTOF II or Bruker MALDI-TOF MS Autoflex speed. Gel permeation chromatography (GPC) was executed with LaboACE LC–5060 equipped with JAIGEL-1HR and JAIGEL-2HR (CHCl3). HPLC purification was performed on the HITACHI HPLC system consisting of the following: pump, L6250; detector, L-3350 RI monitor; column, Senshu-Pak PEGASIL silica SP100; mobile phase, hexane/EtOAc.

Synthesis of trehalose monomycolate analogue

TME (ΔOH): lacking an α-branched alkyl chain

TME (Δbranch, ΔOH): lacking an α-branched alkyl chain and a β-hydroxy group

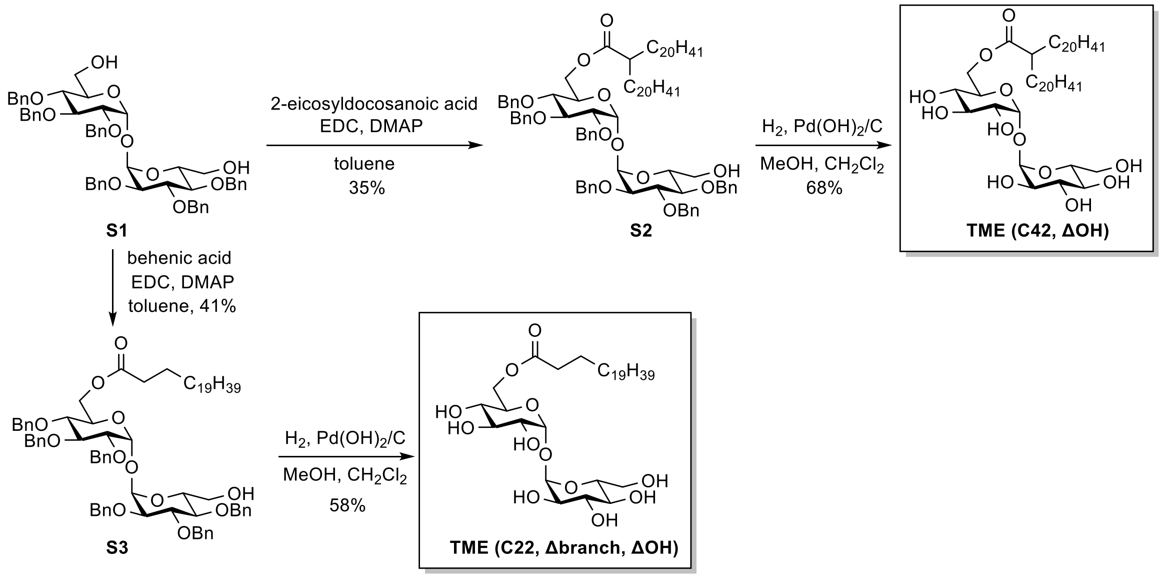

Compound S2

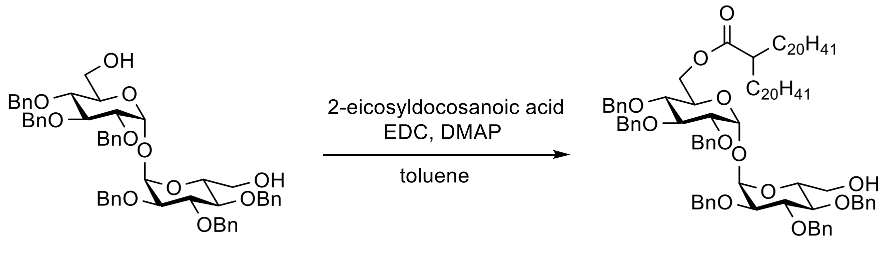

To a solution of compound **S1** (*1, 2*) (92.0 mg, 104 μmol), 2-eicosyldocosanoic acid (*3*) (90.6 mg, 146 μmol, 1.4 equiv.), and DMAP (17.8 mg, 146 μmol, 1.4 equiv.) in toluene (1 mL) was added EDC·HCl (28.0 mg, 146 μmol, 1.4 equiv.). After stirring at 60 °C for 4 h, the reaction mixture was treated with EtOAc (1 mL) and water (3 mL). After

separating layers, the aqueous layer was extracted with EtOAc (2 mL). The combined organic layer was washed with water (5 mL) and brine (5 mL), dried over Na_2SO_4 and concentrated under reduced pressure. The residue was purified by silica gel column chromatography (Yamazen, ULTRAPACK Silica-40B, eluent: MeOH/$CHCl_3$ = 0/1 to 1/99) to give **S2** as a colorless oil (54.3 mg, 35%): $[\alpha]_D^{27}$ +75.04 (c = 0.54, $CHCl_3$); **^{1}H NMR** (500 MHz, $CDCl_3$) δ 7.38–7.25 (m, 30H), 5.18 (d, *J* = 3.7 Hz, 1H), 5.16 (d, *J* = 3.7 Hz, 1H), 5.01 (d, *J* = 11.0 Hz, 1H), 5.00 (d, *J* = 11.0 Hz, 1H), 4.90–4.86 (m, 4H), 4.73 (d, *J* = 11.9 Hz, 1H), 4.72 (d, *J* = 11.9 Hz, 1H), 4.685 (d, *J* = 11.9 Hz, 1H), 4.681 (d, *J* = 11.9 Hz, 1H), 4.65 (d, *J* = 11.0 Hz, 1H), 4.55 (d, *J* = 10.4 Hz, 1H), 4.23 (ddd, *J* = 9.5, 3.4, 2.1 Hz, 1H), 4.20 (dd, *J* = 12.2, 2.1 Hz, 1H), 4.13 (dd, *J* = 12.2, 3.4 Hz, 1H), 4.07 (dd, *J* = 9.5, 9.5 Hz, 1H), 4.06 (dd, *J* = 9.2, 9.2 Hz, 1H), 4.07–4.03 (m, 1H), 3.60–3.53 (m, 6H), 2.33 (m, 1H), 1.60–1.39 (m, 4H), 1.33–1.16 (m, 72H), 0.89 (t, *J* = 6.7 Hz, 6H); **^{13}C NMR** (126 MHz, $CDCl_3$) δ 176.4, 138.9, 138.8, 138.3, 138.1 (2C), 138.0, 128.7–127.5 (30C), 94.0, 93.9, 81.7 (2C), 79.8, 79.7, 78.0, 77.5, 75.9, 75.7, 75.4, 75.2, 73.2, 73.1, 71.4, 69.4, 62.2, 61.7, 45.9, 32.48, 32.46, 32.1 (2C), 29.9–29.8 (26C), 29.71, 29.69, 29.5 (2C), 27.62, 27.57, 22.8 (2C), 14.3 (2C); **HRMS-ESI (*m/z*)** $[M+Na]^+$ calcd for $C_{96}H_{140}NaO_{12}$, 1509.0276; found 1509.0282.

TME (C42, ΔOH)

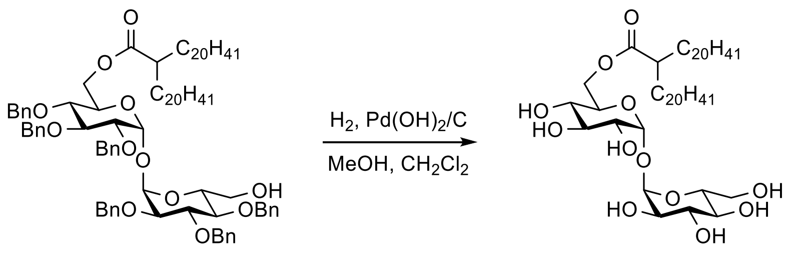

S2 **TME (C42, ΔOH)**

To a solution of **S2** (18.2 mg, 12.2 μmol) in CH_2Cl_2/MeOH (1/1, 3 mL) was added $Pd(OH)_2$/C (5 wt%, 18.2 mg, 6.5 μmol, 53 mol%) under nitrogen atmosphere. The flask was equipped with a hydrogen balloon via a three-way cock and purged with hydrogen. After stirring at room temperature for 18 h, the mixture was filtered through a pad of Celite® and rinsed with CH_2Cl_2/MeOH (1/1). The filtrate was concentrated under reduced pressure. The residue was purified by silica gel column chromatography (eluent: MeOH/$CHCl_3$ = 1/8 to 1/5) to give **TME (C42, ΔOH)** as a white solid (7.9 mg, 68%): **$[\alpha]_D^{28}$** +90.91 (c = 0.20, $CHCl_3$/MeOH 9/1); **^{1}H NMR** (500 MHz, $CDCl_3/CD_3OD$ 9/1) δ 5.05 (d, J = 4.3 Hz, 1H), 5.04 (d, J = 4.3 Hz, 1H), 4.32–4.26 (m, 1H), 4.23 (dd, J = 12.1, 4.0 Hz, 1H), 3.91 (m, 1H), 3.83 (dd, J = 9.5, 9.2 Hz, 1H), 3.82 (dd, J = 9.5, 9.2 Hz, 1H), 3.80–3.73 (m, 2H), 3.63 (dd, J = 12.7, 5.5 Hz, 1H), 3.50–3.44 (m, 2H), 3.33–3.26 (m, 2H), 2.29 (m, 1H), 1.56–1.45 (m, 2H), 1.44–1.34 (m, 2H), 1.33–1.16 (m, 72H), 0.81 (t, J = 7.0 Hz, 6H); **^{13}C NMR** (126 MHz, $CDCl_3/CD_3OD$ 9/1) δ 177.3, 93.7, 93.6, 73.2, 72.8, 72.3, 71.72, 71.65, 70.8, 70.3, 70.2, 62.7, 62.1, 45.7, 32.1, 31.9 (2C), 29.9–29.7 (25C), 29.6 (2C), 29.5 (2C), 29.4 (2C), 27.4, 27.3, 22.7 (2C), 14.1 (2C); **HRMS-MALDI (*m/z*)** $[M+Na]^+$ calcd for $C_{54}H_{104}NaO_{12}$, 967.7425; found 967.74.

Compound S3

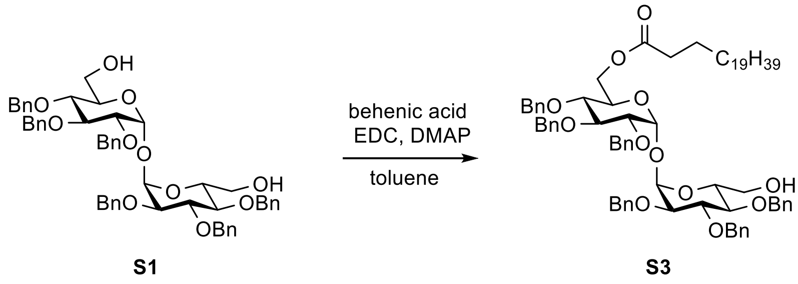

To a solution of compound **S1** (*1, 2*) (235 mg, 266 μmol), behenic acid (127 mg, 373 μmol, 1.4 equiv.), and DMAP (45.5 mg, 373 μmol, 1.4 equiv.) in toluene (2.6 mL) was added EDC•HCl (71.4 mg, 373 μmol, 1.4 equiv.). After stirring at 60 °C for 7 h, the reaction mixture was treated with EtOAc (2 mL) and water (3 mL). After separating the layers, the aqueous layer was extracted with EtOAc (2 mL). The combined organic layer was washed with water (5 mL) and brine (5 mL), dried over Na_2SO_4, and concentrated under reduced pressure. The residue was purified by silica gel column chromatography (Yamazen, ULTRAPACK Silica-40B, eluent: hexane/EtOAc = 4/1 to 3/7) to give **S3** as a colorless oil (132 mg, 41%): **$[\alpha]_D^{24}$** +72.29 (c = 1.0, $CHCl_3$); **^{1}H NMR** (500 MHz, $CDCl_3$) δ 7.39–7.26 (m, 30H), 5.17 (d, *J* = 3.4 Hz, 2H), 5.03 (d, *J* = 11.0 Hz, 1H), 5.01 (d, *J* = 11.0 Hz, 1H), 4.90 (d, *J* = 11.0 Hz, 1H), 4.891 (d, *J* = 11.0 Hz, 1H), 4.888 (d, *J* = 11.0 Hz, 1H), 4.88 (d, *J* = 10.7 Hz, 1H), 4.74 (d, *J* = 11.9 Hz, 1H), 4.72 (d, *J* = 11.9 Hz, 1H), 4.69 (d, *J* = 11.9 Hz, 2H), 4.66 (d, *J* = 11.0 Hz, 1H), 4.54 (d, *J* = 10.7 Hz, 1H), 4.25 (ddd, *J* = 10.1, 3.4, 2.1 Hz, 1H), 4.15 (dd, *J* = 12.1, 3.4 Hz, 1H), 4.11–4.04 (m, 2H), 4.08 (dd, *J* = 9.5, 9.5 Hz, 1H), 4.07 (dd, *J* = 9.5, 9.5 Hz, 1H), 3.61–3.52 (m, 6H), 2.26 (t, *J* = 7.3 Hz, 2H), 1.62–1.54 (m, 2H), 1.50 (t, *J* = 6.7 Hz, 1H), 1.34–1.22 (m,36H), 0.90 (t, *J* = 7.0 Hz, 3H); **^{13}C NMR** (126 MHz, $CDCl_3$) δ 173.6, 138.9, 138.7, 138.3, 138.09, 138.05, 138.0, 128.7–127.6 (30C), 94.2, 94.0, 81.8, 81.7, 79.5 (2C), 77.7, 77.5, 75.8, 75.7, 75.3,

75.2, 73.2, 73.1, 71.4, 69.3, 62.6, 61.7, 34.3, 32.1, 29.9–29.8 (11C), 29.76, 29.6, 29.5, 29.4, 29.3, 25.0, 22.8, 14.3; **HRMS-MALDI (*m/z*)** $[M+Na]^+$ calcd for $C_{76}H_{100}NaO_{12}$, 1227.7112; found 1227.71.

TME (C22, Δbranch, ΔOH)

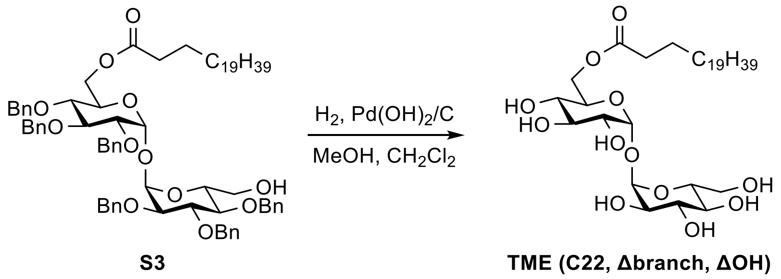

To a solution of **S3** (60.0 mg, 49.8 μmol) in CH_2Cl_2/MeOH (1/1, 3 mL) was added $Pd(OH)_2$/C (5 wt%, 60.0 mg, 21.4 μmol, 43 mol%) under nitrogen atmosphere. The flask was equipped with a hydrogen balloon via a three-way cock and purged with hydrogen. After stirring at room temperature for 18 h, the mixture was filtered through a pad of Celite® and rinsed with MeOH. The filtrate was concentrated under reduced pressure. The residue was washed with ice-cold $CHCl_3$ (0.5 mL) to give **TME (C22, Δbranch, ΔOH)** as a white solid (19.2 mg, 58%): $[\alpha]_D^{23}$ +112.43 (c = 0.20, $CHCl_3$/MeOH 4/1); **^{1}H NMR** (600 MHz, pyridine–d5) δ 7.40 (broad singlet, 1H), 7.27–6.80 (broad multiplet, 5H), 6.32 (broad singlet, 1H), 5.954 (d, *J* = 3.6 Hz, 1H), 5.949 (d, *J* = 3.6 Hz, 1H), 5.12 (ddd, *J* = 9.5, 5.2, 1.9 Hz, 1H), 5.03 (dd, *J* = 11.8, 1.9 Hz, 1H), 4.99 (ddd, *J* = 9.5, 4.6, 2.6 Hz, 1H), 4.86 (dd, *J* = 11.8, 5.2 Hz, 1H), 4.82 (dd, *J* = 9.3, 9.3 Hz, 1H), 4.80 (dd, *J* = 9.3, 9.3 Hz, 1H), 4.49 (dd, *J* = 11.8, 2.6 Hz, 1H), 4.44 (dd, *J* = 11.8, 4.6 Hz, 1H), 4.35 (dd, *J* = 9.5, 9.3 Hz, 1H), 4.32 (dd, *J* = 9.3, 3.6 Hz, 1H), 4.32 (dd, *J* = 9.3, 3.6 Hz, 1H),

4.20 (dd, J = 9.5, 9.3 Hz, 1H), 2.38–2.29 (m, 2H), 1.62 (tt, J = 7.4, 7.4 Hz, 2H), 1.34–1.13 (m, 36H), 0.87 (t, J = 6.9 Hz, 3H); **^{13}C NMR** (151 MHz, pyridine–d5) δ 174.1, 96.1, 96.0, 75.3, 75.2, 74.9, 73.9, 73.8, 72.7, 72.4, 71.9, 64.8, 63.1, 34.8, 32.5, 30.44–30.35 (10C), 30.32, 30.31, 30.2, 30.00, 29.98, 29.8, 25.7, 23.3, 14.7; **HRMS-MALDI (*m/z*)** $[M+Na]^+$ calcd for $C_{34}H_{64}NaO_{12}$, 687.4295; found 687.43.

Synthesis of TMMs

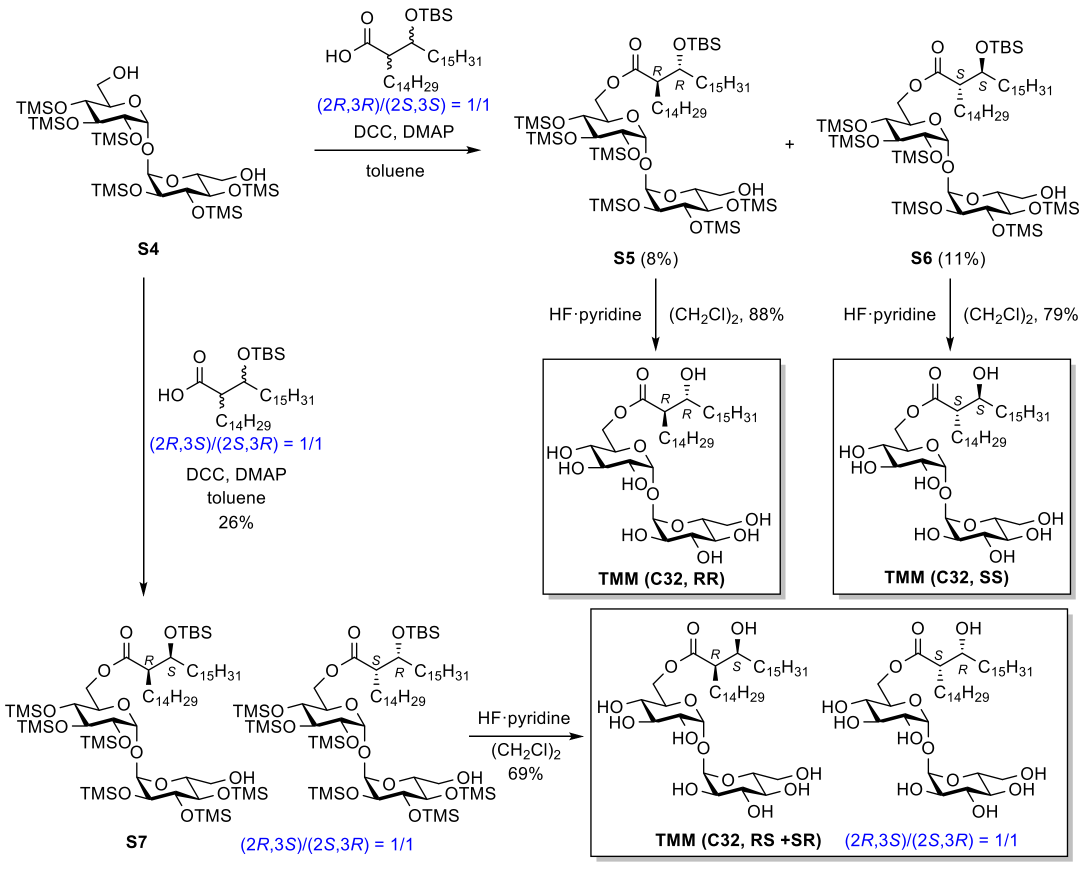

Compound S5 and S6

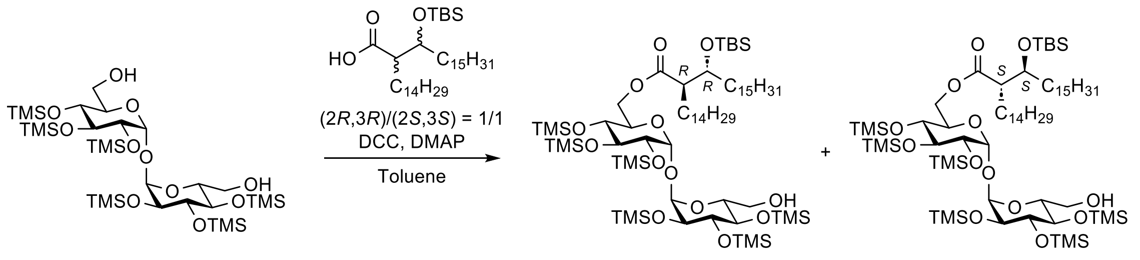

S4 **S5** **S6**

To a mixture of compound **S4** (*4, 5*) (235 mg, 303 μmol), (2RS,3RS)-3-*tert*-butyldimethylsilyloxy-2-tetradecyloctadecanoic acid[5] (185 mg, 303 μmol, 1.0 equiv., diastereomer ratio:1/1), DCC (62.5 mg, 303 μmol, 1.0 equiv.), and DMAP (3.7 mg, 30 μmol, 0.1 equiv.) was added ice-cold toluene (1 mL). The reaction mixture was warmed up to room temperature over 9 h. After stirring at 70 °C for 8 h, the mixture was filtered through a pad of Celite® and rinsed with toluene. The filtrate was concentrated under reduced pressure. The residue was purified by silica gel column chromatography (eluent: hexane/Et_2O = 19/1 to 17/3) and GPC to give the mixture of S**5** and S**6**. The mixture was separated by silica gel column chromatography (Iatrobeads 6RS-8060®, eluent: hexane/Et_2O = 19/1 to 17/3) to give (2*R*,3*R*)-**S5** as a colorless oil (33.9 mg, 8%, less polar) and (2*S*,3*S*)-**S6** as a colorless oil (46.6 mg, 11%, more polar)

Compound **S5**: **$[\alpha]_D^{24}$** +61.86 (c = 0.44, $CHCl_3$); **^{1}H NMR** (500 MHz, $CDCl_3$) δ 4.91 (d, *J* = 3.4 Hz, 1H), 4.84 (d, *J* = 3.1 Hz, 1H), 4.34 (dd, *J* = 11.9, 2.1 Hz, 1H), 4.07 (dd, *J* = 11.9, 4.0 Hz, 1H), 3.98 (ddd, *J* = 9.0, 4.0, 2.1 Hz, 1H), 3.94 (m, 1H), 3.90 (dd, *J* = 9.3, 9.2 Hz, 1H), 3.89 (dd, *J* = 9.5, 9.2 Hz, 1H), 3.84 (ddd, *J* = 9.2, 3.7, 2.8 Hz, 1H), 3.70 (dd, *J* = 11.9, 2.8 Hz, 1H), 3.67 (dd, *J* = 11.9, 3.7 Hz, 1H), 3.48 (dd, *J* = 9.2, 9.0 Hz, 1H), 3.47 (dd, *J* = 9.2, 9.2 Hz, 1H), 3.42 (dd, *J* = 9.3, 3.4 Hz, 1H), 3.38 (dd, *J* = 9.5, 3.1 Hz, 1H), 2.55 (ddd, *J* = 10.6, 5.5, 3.4 Hz, 1H), 1.65–1.55 (m, 2H), 1.50–1.18 (m, 52H), 0.88 (t, *J*

= 6.7 Hz, 6H), 0.87 (s, 9H), 0.16 (s, 9H), 0.150 (s, 9H), 0.145 (s, 9H), 0.141 (s, 9H), 0.139 (s, 9H), 0.11 (s, 9H), 0.051 (s, 3H), 0.047 (s, 3H**)**; **^{13}C NMR** (126 MHz, $CDCl_3$) δ 174.2, 94.7, 94.5, 73.6, 73.5 (2C), 73.03, 72.96, 72.9, 72.1, 71.6, 70.9, 62.6, 61.8, 52.0, 33.6, 32.1 (2C), 30.0, 29.94, 29.9–29.73 (14C), 29.7, 29.5, 28.3 (2C), 26.5, 26.0 (3C), 25.0, 22.9 (2C), 18.2, 14.3 (2C), 1.22 (3C), 1.17 (3C), 1.1 (3C), 1.0 (3C), 0.3 (3C), 0.2 (3C), –4.3, –4.5; **HRMS-MALDI (*m/z*)** $[M+Na]^+$ calcd for $C_{68}H_{146}NaO_{13}Si_7$, 1389.9046; found 1389.90.

Compound **S6**: **$[\alpha]_D^{23}$** +62.92 (c = 0.59, $CHCl_3$); **^{1}H NMR** (500 MHz, $CDCl_3$) δ 4.92 (d, *J* = 3.4 Hz, 1H), 4.85 (d, *J* = 3.1 Hz, 1H), 4.51 (m, 1H), 4.00–3.92 (m, 3H), 3.91 (dd, *J* = 9.2, 8.9 Hz, 1H), 3.89 (dd, *J* = 9.3, 8.9 Hz, 1H), 3.82 (ddd, *J* = 9.2, 4.0, 2.8 Hz, 1H), 3.70 (dd, *J* = 11.8, 2.8 Hz, 1H), 3.66 (dd, *J* = 11.8, 4.0 Hz, 1H), 3.47 (dd, *J* = 9.2, 8.9 Hz, 2H), 3.42 (dd, *J* = 9.3, 3.4 Hz, 1H), 3.36 (dd, *J* = 9.2, 3.1 Hz, 1H), 2.54 (ddd, *J* = 10.4, 6.0, 3.7 Hz, 1H), 1.62–1.53 (m, 1H), 1.52–1.38 (m, 3H), 1.37–1.16 (m, 50H), 0.88 (t, *J* = 6.7 Hz, 6H), 0.86 (s, 9H), 0.16 (s, 9H), 0.155 (s, 9H), 0.151 (s, 9H), 0.145 (s, 9H), 0.139 (s, 9H), 0.11 (s, 9H), 0.05 (s, 3H), 0.03 (s, 3H); **^{13}C NMR** (126 MHz, $CDCl_3$) δ 174.3, 94.5, 94.4, 73.7, 73.5, 73.2, 73.03, 72.99, 72.9, 72.2, 71.6, 71.1, 62.5, 61.8, 52.1, 33.5, 32.1 (2C), 30.0, 29.9–29.8 (15C), 29.7, 29.5 (2C), 28.2, 26.9, 26.0 (3C), 24.7, 22.9 (2C), 18.2, 14.3 (2C), 1.3 (3C), 1.2 (6C), 1.0 (3C), 0.4 (3C), 0.2 (3C), –4.4, –4.6; **HRMS-MALDI (*m/z*)** $[M+Na]^+$ calcd for $C_{68}H_{146}NaO_{13}Si_7$, 1389.9046; found 1389.90.

Compound S7

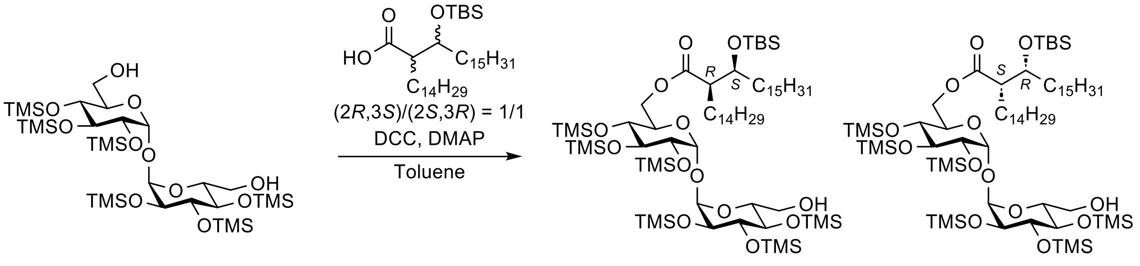

S4 **S7** (2*R*,3*S*)/(2*S*,3*R*) = 1/1

To a mixture of compound **S4** (*4, 5*) (127 mg, 164 μmol), (2RS,3SR)-3-*tert*-butyldimethylsilyloxy-2-tetradecyloctadecanoic acid[5] (100 mg, 164 μmol, 1.0 equiv., diastereomer ratio:1/1), DCC (33.8 mg, 164 μmol, 1.0 equiv.), and DMAP (2.0 mg, 16 μmol, 0.1 equiv.) was added ice-cold toluene (1 mL). The reaction mixture was warmed up to room temperature over 3 h. After stirring at 70°C for 8 h, the mixture was filtered through a pad of Celite® and rinsed with toluene. The filtrate was concentrated under reduced pressure. The residue was purified by silica gel column chromatography (eluent: hexane/Et_2O = 19/1 to 17/3) and GPC (eluent: $CHCl_3$) to give (2*R*,3*S*)/(2*S*, 3*R*)-**S7** as a colorless oil (59.1 mg, 26%, diastereomer ratio:1/1): **$[\alpha]_D^{24}$** +51.20 (c = 0.20, $CHCl_3$); **^{1}H NMR** (500 MHz, $CDCl_3$) δ 4.92 (d, *J* = 3.1 Hz, 1H), 4.89 (d, *J* = 3.1 Hz, 1H), 4.84 (d, *J* = 3.1 Hz, 1H), 4.83 (d, *J* = 3.1 Hz, 1H), 4.61 (dd, *J* = 11.9, 2.1 Hz, 1H), 4.46 (dd, *J* = 11.9, 2.4 Hz, 1H), 4.03 (dd, *J* = 11.9, 3.4 Hz, 1H), 3.99–3.94 (m, 2H), 3.93–3.81 (m, 9H), 3.70 (dd, *J* = 11.6, 2.8 Hz, 2H), 3.66 (dd, *J* = 11.6, 3.1 Hz, 2H), 3.53–3.44 (m, 4H), 3.42 (dd, *J* = 9.5, 3.1 Hz, 1H), 3.41 (dd, *J* = 9.5, 3.1 Hz, 1H), 3.38 (dd, *J* = 9.3, 3.1 Hz, 1H), 3.35 (dd, *J* = 9.5, 3.1 Hz, 1H), 2.53–2.46 (m, 2H), 1.74–1.43 (m, 8H), 1.39–1.15 (m, 100H), 0.89 (s, 9H), 0.882 (s, 9H), 0.879 (t, *J* = 7.0 Hz, 12H), 0.16 (s, 27H), 0.15 (s, 18H), 0.143 (s, 27H), 0.138 (s, 9H), 0.136 (s, 9H), 0.109 (s, 9H), 0.06 (s, 3H), 0.05 (s, 3H), 0.044 (s, 3H), 0.038 (s, 3H); **^{13}C NMR** (126 MHz, $CDCl_3$) δ 174.64, 174.62, 94.7, 94.65

(2C), 94.56, 73.8, 73.6, 73.5 (2C), 73.4, 73.3, 73.04, 73.02, 72.97 (2C), 72.89, 72.86, 72.2, 72.0, 71.6, 71.5, 71.2, 71.0, 62.18, 62.15, 61.82, 61.79, 51.9, 51.7, 35.5, 35.3, 32.1 (4C), 30.2, 30.1, 30.0, 29.9–29.74 (30C), 29.7, 29.6, 29.5 (4C), 28.8, 28.0, 27.7, 26.1 (6C), 24.4, 24.2, 22.9 (4C), 18.29, 18.27, 14.3 (4C), 1.23 (6C), 1.17 (6C), 1.1 (6C), 1.0 (6C), 0.4 (3C), 0.34 (3C), 0.26 (3C), 0.2 (3C), –4.0, –4.1, –4.3, –4.4; **HRMS-MALDI (*m/z*)**: $[M+Na]^+$ calcd for $C_{68}H_{146}NaO_{13}Si_7$, 1389.9046; found 1389.90.

TMM (C32, *RR*)

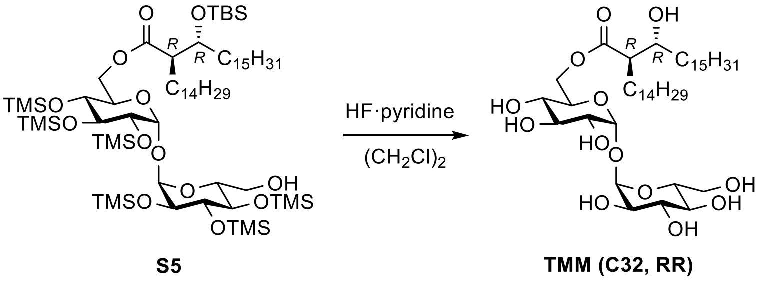

To a solution of compound **S5** (11.0 mg, 8.04 μmol) in $(CH_2Cl)_2$ (0.3 mL) was added 70% HF•pyridine (43 μL) at room temperature. After stirring at room temperature for 11 h, the resulting mixture was poured into a saturated aqueous $NaHCO_3/CHCl_3$ solution (1/1, 1 mL). The water layer was extracted with $CHCl_3$ (2 x 1 mL). The combined organic layer was dried over Na_2SO_4 and concentrated under reduced pressure. The residue was purified by silica gel column chromatography (Iatrobeads 6RS-8060®, eluent: $CHCl_3$/MeOH = 9/1 to 5/1) to give **TMM (C32, *RR*)** as a white solid (5.8 mg, 88%).

TMM (C32, *SS*)

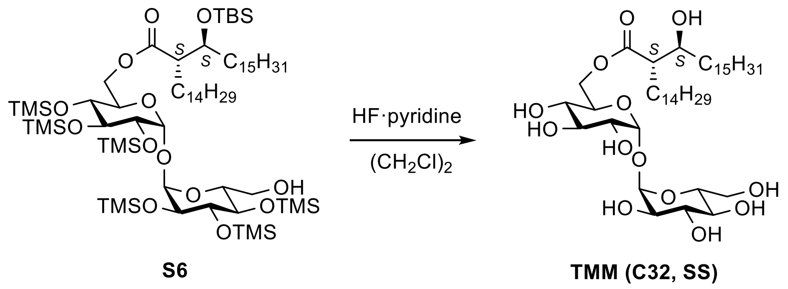

To a solution of compound **S6** (12.4 mg, 9.06 μmol) in $(CH_2Cl)_2$ (0.3 mL) was added 70% HF▪pyridine (49 μL) at room temperature. After stirring at room temperature for 11 h, the resulting mixture was poured into a saturated aqueous $NaHCO_3/CHCl_3$ solution (1/1, 1 mL). The water layer was extracted with $CHCl_3$ (2 x 1 mL). The combined organic layer was dried over Na_2SO_4 and concentrated under reduced pressure. The residue was purified by silica gel column chromatography (Iatrobeads 6RS-8060®, eluent: $CHCl_3$/MeOH = 9/1 to 5/1) to give **TMM (C32, *SS*)** as a white solid (5.9 mg, 79%).

TMM (C32, *RS* +*SR*)

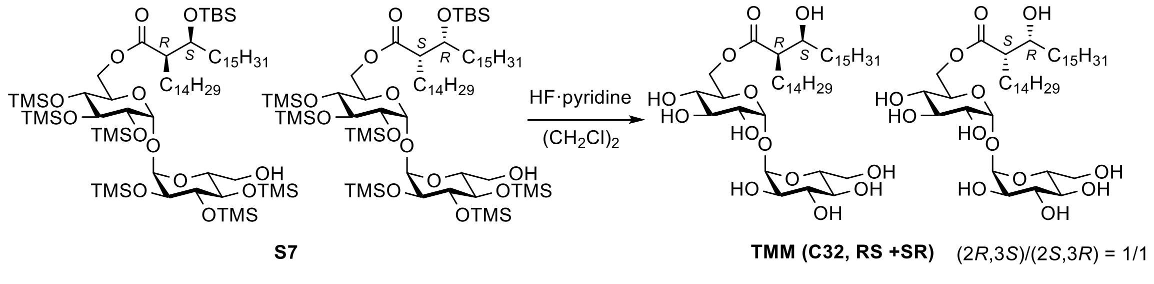

To a solution of compound S7 (23.8 mg, 17.4 μmol) in $(CH_2Cl)_2$ (0.3 mL) was added 70% HF▪pyridine (94 μL) at room temperature. After stirring at room temperature for 11 h, the resulting mixture was poured into a saturated aqueous $NaHCO_3/CHCl_3$ solution (1/1, 1 mL). The water layer was extracted with $CHCl_3$ (2 x 1 mL). The combined organic layer was dried over Na_2SO_4 and concentrated under reduced pressure. The residue was

purified by silica gel column chromatography (Iatrobeads 6RS-8060®, eluent: $CHCl_3$/MeOH = 9/1 to 5/1) to give **TMM (C32, *RS*+*SR*)** as a white solid (9.8 mg, 69%).

Large scale chemical synthesis of TMM

Synthesis of the trehalose precursor S11

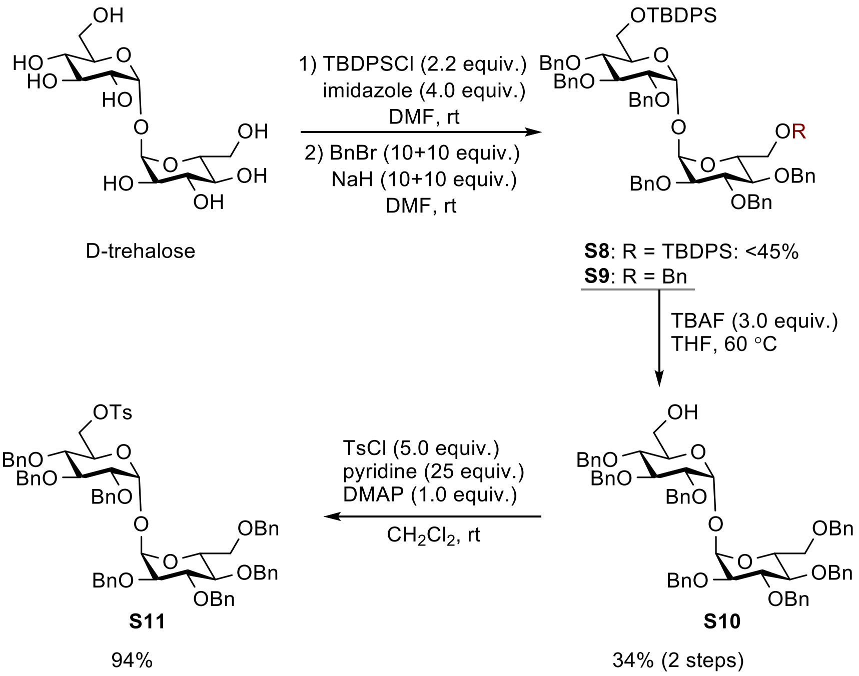

Compound S11

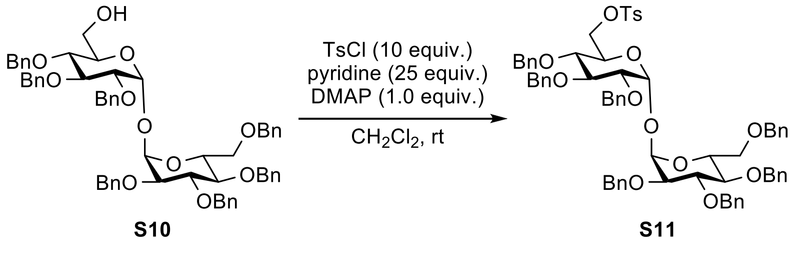

To a solution of **S10** (***6***) (85.6 mg, 88.0 µmol), DMAP (10.7 mg, 88.0 µmol, 1.0 equiv.) and pyridine (178 µL, 2.20 mmol, 25 equiv.) in dichloromethane (880 µL) was added TsCl (168 mg, 880 µmol, 10 equiv.). After stirring for 18 h at room temperature, the resulting solution was treated with aqueous saturated $NaHCO_3$ (2 mL) and extracted with EtOAc (20 mL). After separating layers, the organic layer was washed with brine (5 mL),

dried over Na_2SO_4, and concentrated under reduced pressure. The residue was purified by silica gel column chromatography (Biotage Sfär Silica HC Duo 10 g, hexane/EtOAc = 92/8 to 71/29) to give **S11** (83.5 mg, 74.1 μmol, 84%) as a colorless oil.

Compound **S11**: **$[\alpha]_D^{18}$** +64.93 (*c* 2.65, $CHCl_3$); **^{1}H NMR** (500 MHz, $CDCl_3$): δ 7.68 (d, *J* = 8.2 Hz, 2H), 7.35–7.18 (m, 33H), 7.13–7.08 (m, 4H), 5.11–5.08 (m, 2H), 4.97 (d, *J* = 10.7 Hz, 1H), 4.95 (d, *J* = 11.0 Hz, 1H), 4.85 (d, *J* = 11.0 Hz, 1H), 4.82–4.76 (m, 3H), 4.69 (d, *J* = 11.9 Hz, 1H), 4.66–4.60 (m, 2H), 4.57 (d, *J* = 11.9 Hz, 1H), 4.53 (d, *J* = 12.2 Hz, 1H), 4.45 (d, *J* = 10.7 Hz, 1H), 4.39–4.35 (m, 2H), 4.15–4.08 (m, 2H), 4.00–3.92 (m, 3H), 3.80 (dd, *J* = 10.7, 1.8 Hz, 1H), 3.65 (dd, *J* = 9.5, 8.9 Hz, 1H), 3.55 (dd, *J* = 9.8, 3.7 Hz, 1H), 3.53–3.45 (m, 3H), 3.36 (dd, *J* = 10.7, 1.8 Hz, 1H), 2.37 (s, 3H); **^{13}C NMR** (126 MHz, $CDCl_3$): δ 144.9, 138.9, 138.8, 138.4, 138.2, 138.1, 138.0, 137.9, 132.9, 129.9 (2C), 128.50–128.48 (16C), 128.1 (7C), 128.0 (2C), 127.9 (2C), 127.8 (2C), 127.68–127.66 (4C), 127.5 (2C), 127.4 (2C), 94.7, 94.3, 81.9, 81.6, 79.5, 79.3, 77.8, 77.0, 75.70, 75.67, 75.24, 75.17, 73.6, 73.1, 72.8, 70.9, 68.9, 68.3, 68.2, 21.8; **HRMS-ESI (*m/z*)**: $[M+Na]^+$ calcd for $C_{68}H_{70}NaO_{13}S$, 1149.4435; found 1149.4412

Compound S12

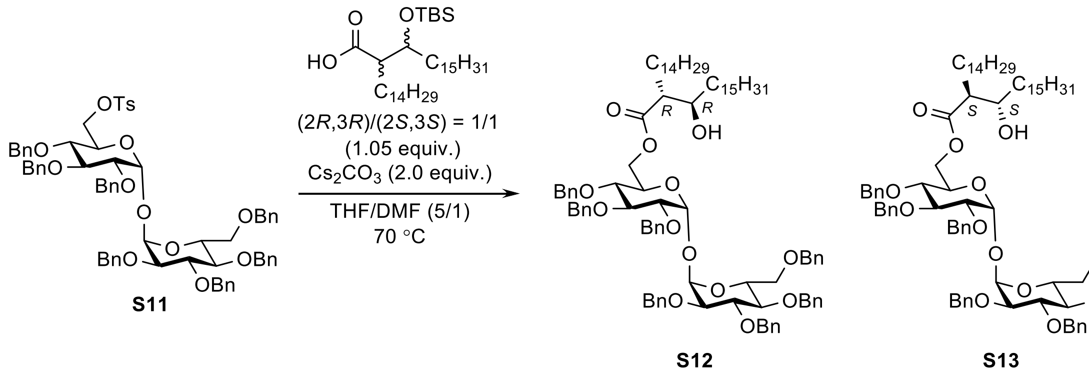

To a solution of **S11** (41.7 mg, 36.5 μmol) and (2*RS*,3*RS*)-3-tert-butyldimethylsilyloxy-2-tetradecyloctadecanoic acid(*5*) (19.1 mg, 38.4 μmol, 1.05 equiv.) in THF/DMF (360 μL, 5:1) was added Cs_2CO_3 (23.8 mg, 73.1 μmol, 2.0 equiv.) at room temperature. After stirring for 48 h at 70 °C, the resulting solution was poured into a mixture of Et_2O (5 mL) and water (5 mL). After separating layers, the organic layer was washed with brine (3 mL), dried over Na_2SO_4, and concentrated under reduced pressure. The residue was purified by MPLC (YAMAZEN, Ultrapack, silica-40B, hexane/EtOAc 97/3 to 85/15). Further separation was carried out by HPLC (Senshu Pack PEGASIL silica SP100 ϕ 20, hexane/EtOAc = 85/15) to give **S12** (12.6 mg, 8.90 μmol, 24%) as a colorless oil and **S13** (15.4 mg, 10.6 μmol, 29%) as a colorless oil.

Compound **S12**: **$[\alpha]_D^{18}$** +76.24 (*c* 0.11, $CHCl_3$); **^{1}H NMR** (500 MHz, $CDCl_3$): δ 7.36–7.20 (m, 33H), 7.14–7.11 (m, 2H), 5.21 (d, *J* = 3.7Hz, 1H), 5.20 (d, *J* = 3.1 Hz, 1H), 5.00 (d, *J* = 10.7 Hz, 1H), 4.98 (d, *J* = 10.7 Hz, 1H), 4.90–4.84 (m, 3H), 4.81 (d, *J* = 10.7 Hz, 1H), 4.73 (d, *J* = 11.9 Hz, 1H), 4.70–4.66 (m, 3H), 4.55 (d, *J* = 10.1 Hz, 1H), 4.53 (d, *J* = 12.2 Hz, 1H, 4.47 (d, *J* = 10.7 Hz, 1H), 4.38 (d, *J* = 12.2 Hz, 1H), 4.25 (ddd, *J* = 10.1,

2.8, 2.1 Hz, 1H), 4.21 (dd, *J* = 12.2, 2.1 Hz, 1H), 4.17–4.11 (m, 2H), 4.06 (dd, *J* = 9.2, 9.2 Hz, 1H), 4.01 (dd, *J* = 9.5, 9.2 Hz, 1H), 3.67 (dd, *J* =10.1, 9.2 Hz, 1H), 3.65–3.61 (m, 1H), 3.60 (dd, *J* = 9.5, 3.7 Hz, 1H), 3.56–3.51 (m, 2H), 3.50 (dd, *J* = 10.7, 3.4 Hz, 1H), 3.36 (dd, *J* = 10.7, 1.8 Hz, 1H), 2.50–2.30 (m, 2H), 1.69–1.51 (m, 4H), 1.47–1.16 (m, 50H), 0.91–0.87 (m, 6H); **^{13}C NMR** (126 MHz, $CDCl_3$): δ 175.4, 139.0, 138.8, 138.5, 138.2, 138.1 (2C), 138.0, 128.60 (2C), 128.58 (2C), 128.54 (2C), 128.51–128.44 (8C), 128.13–128.05 (8C), 128.03, 128.00 (2C), 127.9, 127.81 (2C), 127.76, 127.66 (2C), 127.6 (2C), 127.4 (2C), 94.5, 94.1, 81.9, 81.7, 79.8, 79.5, 78.0, 77.8, 75.8, 75.7, 75.4, 75.2, 73.6, 73.1, 72.9, 72.3, 70.9, 69.1, 68.3, 62.5, 51.4, 35.6, 32.1 (2C), 29.90–29.74 (16C), 29.6 (2C), 29.5 (2C), 27.7, 25.9, 22.8 (2C), 14.3 (2C); **HRMS-ESI (*m/z*)**: $[M+Na]^+$ calcd for $C_{93}H_{126}NaO_{13}$, 1473.9096; found 1473.9079

Compound **S13**: **$[\alpha]_D^{18}$** +61.05 (*c* 0.22, $CHCl_3$); **^{1}H NMR** (500 MHz, $CDCl_3$): δ 7.36–7.20 (m, 33H), 7.14–7.10 (m, 2H), 5.20 (d, *J* = 3.7 Hz, 1H), 5.19 (d, *J* = 3.7 Hz, 1H), 4.99 (d, *J* = 11.0 Hz, 1H), 4.98 (d, *J* = 10.7 Hz, 1H), 4.89–4.84 (m, 3H), 4.80 (d, *J* = 10.7 Hz, 1H), 4.72 (d, *J* = 11.9 Hz, 1H), 4.71–4.65 (m, 3H), 4.55 (d, *J* = 10.7 Hz, 1H), 4.53 (d, *J* = 11.9 Hz, 1H), 4.46 (d, *J* = 10.7 Hz, 1H), 4.37 (d, *J* = 12.2 Hz, 1H), 4.24 (ddd, *J* = 10.1, 2.4, 2.1 Hz, 1H), 4.20 (dd, *J* = 12.2, 2.1 Hz, 1H), 4.16–4.11 (m, 2H), 4.04 (dd, *J* = 9.2, 9.2 Hz, 1H), 4.01 (dd, *J* = 9.5, 9.2 Hz, 1H), 3.67 (dd, *J* = 9.8, 9.2 Hz, 1H), 3.66–3.61 (m, 1H), 3.60 (dd, *J* = 9.5, 3.7 Hz, 1H), 3.58–3.51 (m, 2H), 3.48 (dd, *J* = 11.0, 3.4 Hz, 1H), 3.34 (dd, *J* = 11.0, 1.8 Hz, 1H), 2.44–2.38 (m, 2H), 1.69–1.51 (m, 4H), 1.48–1.17 (m, 50H), 0.91–0.85 (m, 6H); **^{13}C NMR** (126 MHz, $CDCl_3$): δ 175.2, 139.0, 138.8, 138.5,

138.2, 138.1 (2C), 138.0, 128.64–128.44 (14C), 128.15–128.04 (9C), 128.01 (2C), 127.9, 127.82 (2C), 127.77, 127.7 (2C), 127.6 (2C), 127.4 (2C), 94.6, 94.2, 81.9, 81.7, 79.9, 79.5, 77.9, 77.8, 75.9, 75.7, 75.4, 75.2, 73.7, 73.2, 73.0, 72.3, 70.9, 69.2, 68.3, 62.3, 51.7, 35.7, 32.1 (2C), 29.90–29.73 (16C), 29.67, 29.65, 29.5 (2C), 27.5, 25.9, 22.9 (2C), 14.3 (2C); **HRMS-ESI (*m/z*)**: $[M+Na]^+$ calcd for $C_{93}H_{126}NaO_{13}$, 1473.9096; found 1473.9102

TMM (C32, *RR*)

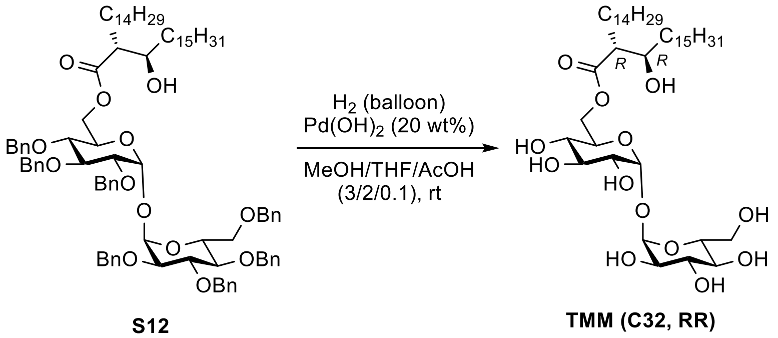

To a solution of **S12** (153 mg, 106 μmol) in methanol (3.0 mL), THF (2.0 mL), and AcOH (0.10 mL) was added $Pd(OH)_2/C$ (30 mg, 20 wt%) under argon atmosphere. The flask was equipped with a hydrogen balloon via a three-way cock and purged with hydrogen. After stirring for 24 h at room temperature, the mixture was filtered through a pad of Celite®, and the filtrate was concentrated. The residue was purified by silica gel column chromatography (Iatrobeads 6RS-8060®, $CHCl_3$/MeOH 10/1 to 2/1) to give **TMM (C32, *RR*)** (79.8 mg, 97.2 μmol, 92%) as a pale yellow solid.

The ^{1}H-NMR spectrum of the product obtained here exhibited somewhat different chemical shifts compared to previously reported data(*7*), likely due to variations in

solvent composition and compound concentration. However, the ^{13}C-NMR spectrum showed a general agreement. Consequently, it can be concluded that the product is **TMM (C32, *RR*)**.

References

1. S. R. Gilbertson, C.-W. T. Chang, Synthesis of New Disugar Phosphine Ligands and Their Use in Asymmetric Hydrogenation. *The Journal of Organic Chemistry* **60**, 6226-6228 (2002).
2. A. A. Khan *et al.*, Long-chain lipids are required for the innate immune recognition of trehalose diesters by macrophages. *Chembiochem* **12**, 2572-2576 (2011).
3. Y.-Q. Zheng *et al.*, Influence of solution-state aggregation on conjugated polymer crystallization in thin films and microwire crystals. *Giant* **7**, (2021).
4. V. A. Sarpe, S. S. Kulkarni, Synthesis of maradolipid. *J Org Chem* **76**, 6866-6870 (2011).
5. A. K. Datta, K. Takayama, M. A. Nashed, L. Anderson, An improved synthesis of trehalose 6-mono- and 6,6'-di-corynomycolates and related esters. *Carbohydr Res* **218**, 95-109 (1991).
6. K. M. Backus *et al.*, Uptake of unnatural trehalose analogs as a reporter for Mycobacterium tuberculosis. *Nat Chem Biol* **7**, 228-235 (2011).
7. T. Ikazaki *et al.*, Ligand-Controlled Stereoselective Synthesis and Biological Activity of 2-Exomethylene Pseudo-glycoconjugates: Discovery of Mincle-Selective Ligands. *Angew Chem Int Ed Engl* **62**, e202302569 (2023).
