# Supplementary figures and images for: A conserved human CD4+ T cell subset recognizing the mycobacterial adjuvant trehalose monomycolate
Source: J Clin Invest. 2024 Dec 24;135(6):e185443. doi: 10.1172/JCI185443 (PMC11910211; doi:10.1172/JCI185443)

Supplemental Figure 3B

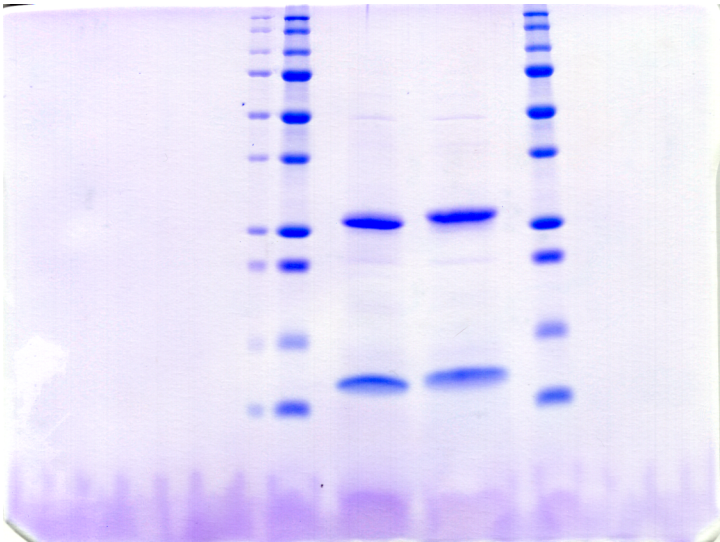

Supplement: Unedited blot and gel images [file jci-135-185443-s252.pdf]
